# Prognostic gene expression signatures of breast cancer are lacking a sensible biological meaning

**DOI:** 10.1038/s41598-020-79375-y

**Published:** 2021-01-08

**Authors:** Kalifa Manjang, Shailesh Tripathi, Olli Yli-Harja, Matthias Dehmer, Galina Glazko, Frank Emmert-Streib

**Affiliations:** 1 Predictive Society and Data Analytics Lab, Tampere University, Tampere, Korkeakoulunkatu 10, 33720 Tampere, Finland; 2 Computational Systems Biology, Tampere University, Tampere, Korkeakoulunkatu 10, 33720 Tampere, Finland; 3 Institute for Systems Biology, Seattle, WA USA; 4 Steyr School of Management, University of Applied Sciences Upper Austria, 4400 Steyr Campus, Wels, Austria; 5 College of Artificial Intelligence, Nankai University, Tianjin, 300350 China; 6 Department of Biomedical Computer Science and Mechatronics, UMIT-The Health and Life Science University, 6060 Hall in Tyrol, Innsbruck, Austria; 7 Department of Biomedical Informatics, University of Arkansas for Medical Sciences, Little Rock, USA; 8 Institute of Biosciences and Medical Technology, Tampere University, Tampere, Korkeakoulunkatu 10, 33720 Tampere, Finland

**Keywords:** Breast cancer, Microarrays, Statistical methods, Cancer, Computational biology and bioinformatics, Systems biology, Mathematics and computing

## Abstract

The identification of prognostic biomarkers for predicting cancer progression is an important problem for two reasons. First, such biomarkers find practical application in a clinical context for the treatment of patients. Second, interrogation of the biomarkers themselves is assumed to lead to novel insights of disease mechanisms and the underlying molecular processes that cause the pathological behavior. For breast cancer, many signatures based on gene expression values have been reported to be associated with overall survival. Consequently, such signatures have been used for suggesting biological explanations of breast cancer and drug mechanisms. In this paper, we demonstrate for a large number of breast cancer signatures that such an implication is not justified. Our approach eliminates systematically all traces of biological meaning of signature genes and shows that among the remaining genes, surrogate gene sets can be formed with indistinguishable prognostic prediction capabilities and opposite biological meaning. Hence, our results demonstrate that none of the studied signatures has a sensible biological interpretation or meaning with respect to disease etiology. Overall, this shows that prognostic signatures are black-box models with sensible predictions of breast cancer outcome but no value for revealing causal connections. Furthermore, we show that the number of such surrogate gene sets is not small but very large.

## Introduction

Since the inception of high-throughput technologies the goal has been to utilize such experimental devices not only for obtaining a better elucidation of biology but to translate this knowledge into the clinical practice^1,2^. One particular example for such an application are prognostic studies based on gene expression data^3–5^. In general, the goal of such studies is to select a, preferably small, number of genes as features, called a signature, and to utilize these for predicting the course of a disease or outcome of patients represented by gene expression profiles. The prognostic value of such predictions is quantitatively assessed via a survival analysis allowing to perform a statistical test for detecting differences in different patient groups with respect to ’time to event’ information. Due to the generality of ’event’, which cannot only be death but also relapse or development of metastasis or organ rejection, prognostic studies are relevant for nearly all patient-related medical investigations. Due to the importance of prognostic studies for clinical applications and their general complexity, statistical aspects of this problem have attracted much attention in the literature. For instance, in^6^ the authors addressed the stability of the selection of prognostic predictors for various cancer types. They found that the size of the training data and the patients in it has a crucial effect on this. The same problem has been studied for breast cancer in^7^ and the authors found that thousands of patient samples are needed for achieving an overlap of 50% between two predictive sets of genes. Such problems have been confirmed in many comparative investigations of feature selection mechanisms, see, e.g.,^8–10^.

For breast cancer, an early study of a prognostic gene expression signature is from^11^. The authors used a 70-gene signature to distinguish good prognosis from bad prognosis groups of patients with stage I or II breast cancer. The outcome of this influential paper sparked many follow-up investigations. For instance, in^12^ a 76-gene signature was used predicting development of distant metastases within 5 years of lymph-node-negative primary breast cancer or in^13^ an invasiveness signature of a 186-gene signature was used for predicting overall and metastasis-free survival. It is important to note that for all such studies not only the predictive outcome is of value but also the interpretational biological meaning of the used signatures^14^. Specifically, it has been stated in^7^ that “A reliable set of predictive genes also will contribute to a better understanding of the biological mechanism of metastasis”. This assumption is not limited to the above problem but widely believed to be true in the genomics and translational medicine community. The main purpose of this paper is to refute this assumption.

Our study is different from the above mentioned ones with respect to the following aspects. First, we do not introduce a new procedure for selecting signature genes. Instead, we provide an analysis of previously introduced signatures with respect to their biological meaning. Second, we do not introduce a new validation method because all studied signatures have been previously validated, although we are using an independent validation data set for our study. Third, we do not aim to improve the quality of different prognostic signatures, although we utilize a more stringent statistical assessment, including conservative multiple testing corrections, compared to previous studies. Fourth, we do also not establish a connection between a prognostic signature and disease etiology shedding light on the underlying molecular and cell biological mechanisms. Instead, we investigate the prognostic benefit of random gene sets having a constrained biological meaning. The main purpose of this paper is to systematically demonstrate that sensible prognostic signatures of breast cancer outcome do not have a sensible biological meaning with respect to disease etiology. This is accomplished via *constrained-sampling*, a restricted resampling procedure for constructing random gene sets, which we introduce in this paper.

A central aspect of our constrained-sampling analysis is based on the definition of biological meaning of a set of genes. For this, we are using two different commonly utilized approaches. The first is centered around the meaning of individual ‘genes’ and the second is based on ‘biological processes’. For the gene-based definition of biological meaning, we follow a Mendelian-view whereas for the biological process-based definition representing a systems-view^15^, we utilize Gene Ontology (GO)^16^ and its underlying hierarchically organized GO-terms in the form of a directed acyclic graph (DAG).

This paper is organized as follows. In the next section, we describe the underlying methodology and the used data. Then we present our results and discuss our findings. This paper finishes with concluding remarks.

## Methods

In this section, we provide information about the data and methods used for our analysis.

### Gene expression data and BM signatures

Our analysis makes use of two sources of data—gene expression data and sets of breast cancer gene signatures from 48 published studies. For the gene expression data we use two different data sets publicly available. The first gene expression dataset (in the following called NKI breast cancer) is accessible from^17^ and it contains 295 breast cancer samples from the Netherlands Cancer institute (a.k.a NKI) cohort. The data were generated by^11^. The gene expression dataset consists of 13108 genes and each sample corresponds to one patient. All patients had stage I or II breast cancer. The dataset is complemented by information about the development of metastases which has been used to indicate an ’event’ for survival analysis. The second gene expression dataset (in the following called SWE breast cancer) is from Gene Expression Omnibus (GSE96058)^18^. It contains 30865 genes and samples of the subtypes Basal (360), Her2 (348), LumA (1709), LumB (767) and Normal (225). The data were FPKM normalized and log transformed. The 48 biomarker (BM) signatures we use for our analysis were compiled in^17^. The number of genes in each signature varies, but all the biomarkers together contain 8106 genes. For the NKI gene expression data 5350 genes are present and for the SWE data 5060 genes.

### Outcome association

**Figure 1:**
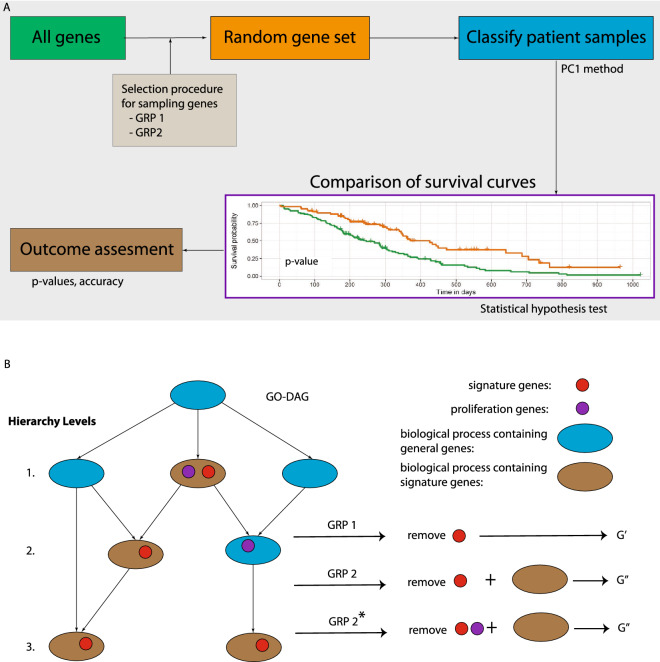
(**A**) Shown is a flowchart of all steps involved in our analysis. (**B**) Visualization of the underlying ideas of GRP 1 and GRP 2. The resulting gene sets $G'$ and $G''$ are used for sampling random gene sets.

For assessing the prognostic value of gene sets, we perform a survival analysis. Specifically, we perform Kaplan Meier estimates of survival curves and compare these with a Mantel–Haenszel test^19^. Hence, each comparison is characterized by a p-value resulting from such a hypothesis test. The categorization of patients is achieved by the PC1 method, described below. This method separates the patients according to specified gene set. This means that the resulting survival analysis is a function of the gene set used to categorize patients. In Fig. 1, we show an overview of the individual steps involved in our analysis. Overall, our analysis consists of three main steps. First, selection/construction of a random gene set, second, classification of patient samples and, third, performing a survival analysis.

In the next two sections, we specify two different gene removal procedures (GRP) for constructing random gene sets. These procedures implement a constrained-sampling for two different views on biology, a Mendelian-view based on genes (GRP 1) and a systems-view based on biological processes (GRP 2 and GRP 2*).

#### Gene removal procedure 1

For this analysis, we investigate the prediction capabilities of random gene sets, $RGS_i$, whereas the genes in $RGS_i$ are randomly sampled from the set $G_i' = G \setminus BM_i$. Here *G* corresponds to the total number of genes in our breast cancer data set and $BM_i$ is the BM signature of study *i*, for $i \in \{1, \dots , 48\}$. The number of genes sampled per random signature is the same as in $BM_i$, i.e., $|RGS_i|=|BM_i|$. We repeat this sampling 1000 times for each study with and without a Bonferroni correction. From numerical analyses we found that increasing the number of repeats does not lead to different results. In total we study 96, 000 random gene sets that have been constructed in this way. Details of this gene removal process are described as follows:

1. *G* : total number of genes in our breast cancer dataset.
2. $BM_i : \{g_1,\ldots ,g_m\}$. $BM_i$ is the gene signature *i* (*i* range from 1 to 48) and $g_1,\ldots ,g_m$ are the genes in the corresponding signature.
3. For each biomarker set *i*:
  - Removing biomarker genes in signature $BM_i$ from *G*. This gives a new set of genes $G_i'$ with $G_i'$ = $G \setminus BM_i$.
  - From $G'$, we sample new sets of biomarker genes of size $|BM_i|$ and perform the prognostic task. This is repeated 1000 times for each study *i*.
  - Application of a Bonferroni correction to the p-values.
  - Assessing the performance for a significance level of $\alpha$.

Overall, gene removal procedure 1 constructs random gene sets by removal of BM signatures. If a random gene set has a significant p-value, we call it a *surrogate gene set* because it has the same prognostic prediction capabilities as a BM signature and hence it is a surrogate for this.

#### Gene removal procedure 2

For this analysis, we do not only remove BM signatures, but we remove also genes that belong to the same biological processes as the genes in the BM signatures. Due to the fact that according to the gene ontology (GO) database^16^ the biological processes are hierarchically organized, we approach this analysis iteratively by removing successively genes of biological processes on the same hierarchy level^20^. Details of this gene removal process are described as follows:

- 1. *G* : total number of genes in our breast cancer dataset.
- 2. $BM_i : \{g_1,\ldots ,g_m\}$. $BM_i$ is the gene signature *i* (*i* range from 1 to 48) and $g_1,\ldots ,g_m$ are the genes in the corresponding signature.
- 3. Removing biomarker genes in signature $BM_i$ from *G*. This gives a new set of genes $G_i'$ with $G_i'$ = $G \setminus BM_i$.
- $3^{*}$ Optional step: Removing proliferation genes in *PG* from *G*. This gives a new set of genes $G_i'^*$ with $G_i'^*$ = $G_i' \setminus PG$.
- 4. Mapping of the genes in $BM_i$ to GO-terms and the corresponding hierarchy levels. This gives:
  1 $$\begin{aligned} BM_i = \{g_1,\ldots ,g_m\} \rightarrow \{(GO_1, L_1), \ldots ,(GO_t, L_t)\} . \end{aligned}$$
  Note, each gene can be connected to more than one GO-term. For this reason $m \le t$.
- 5. Ranking of the GO-terms in descending order with respect to the hierarchy levels.
- 6. For each biomarker set *i*: Loop-over the hierarchy levels *l* in descending order, i.e., for $l \in \{L_{max}(i),\ldots ,L_{min}(i)\}$. Here $L_{max}(i)$ is the highest hierarchy level of biomarker set *i* and $L_{min}(i)$ is the lowest hierarchy level.
  - Delete all the genes associated with GO-terms on level *l*. This results in a new gene set given by $G''$= $G' \setminus D$, where *D* is the set of genes having GO-terms on level *l*.
  - From $G''$, we sample new sets of biomarker genes of size $|BM_i|$ and perform the prognostic task. This is repeated 1000 times for each hierarchy level *l*.
  - Application of a Bonferroni correction to the p-values.
  - Assessing the performance for a significance level of $\alpha$.
  - Set $G' = G''$. Stop if $l=L_{min}(i)$ or $|G''|< |BM_i|$.

In the above procedure, the set *PG* is the gene set consisting of genes related to proliferation. The genes in *PG* have been defined in^21^ and consist of the signature genes of Whitfield^22^ and meta-PCNA^17^. In total *PG* contains 664 genes. Step 3* is an optional step that removes additionally proliferation genes. When step 3* is used, we call the procedure GRP 2*, whereas when step 3* is not used, we call the procedure GRP 2.

Put simply, procedure GRP 2 removes first all biomarker genes (see step 3) and then iteratively removes genes belonging to the same biological processes as the signature genes (see step 6) from the highest hierarchy level $L_{max}$ to the lowest hierarchy level $L_{min}$. That means at the end a set of genes $G''$ is obtained that contains neither signature genes nor genes the belong to the same biological processes as the signature genes regardless of the hierarchy level. Results for $G''$ for intermediate hierarchy levels *l* contain a certain overlap with biological processes as indicated by *l*. All sets $G''$ are treated in a similar way, i.e., the prognostic task is performed and assessed.

We assess the prediction results again by the p-values from the survival analysis. In addition, we determine the accuracy of predictions by declaring significant p-values as true positives (TPs) and non-significant results as false negatives (FNs). This allows the estimation of accuracy values, i.e., $Acc = (TP + TN)/(TP + TN + FP + FN)$ by $Acc=TP/FN$^23^. These evaluations are obtained for each hierarchy level.

Overall, gene removal procedure 2 constructs random gene sets by removal of BM signatures and biological process related genes. Also here a random gene set with a significant p-value is call a *surrogate gene set*. In Fig. 1 B, a visualization of GRP 1, GRP 2 and GRP 2$^*$ is shown.

### Categorize patient samples

For categorizing the samples of the patients, the PC1 stratification method is used. This method is based on a principal component analysis (PCA). The principal component analysis is a dimensionality reduction technique (this involves reducing the size of the data set). The goal is to transform large data set into smaller ones. This method trades a little accuracy for simplicity, thus achieving interpretability as well as minimal loss of information. Using the “prcomp” function available in R, the first principal component (PC1) of the signature is derived. The patients are then divided into two groups according to the median of the PC1. Specifically, a sample is categorized as group $-1$ if the PC1 is below the median value and as group $+1$ if the PC1 is above the median value.

For this analysis, a gene expression matrix of the form $X \in \mathbb {R}^m \times \mathbb {R}^n$ is used whereas *m* is the number of genes and *n* is the number of samples. Importantly, *m* corresponds to a particular gene set and not all genes available. Above, we described two different procedures for constructing such gene sets. Other sets we use for our analysis are the BM signatures themselves.

### Survival analysis

For assessing the prognostic value of gene sets, we perform a survival analysis. Specifically, we perform Kaplan Meier estimates of survival curves and compare these with a Mantel–Haenszel test^19^. Hence, each comparison is characterized by a p-value resulting from such a hypothesis test. The categorization of patients is achieved by the PC1 method, described above. This method separates the patients according to a specified gene set. Therefore, the resulting survival analysis depends on this gene set.

### Definition: biological meaning

In this paper we use the term ‘biological meaning’ in a well-defined way. This definition is based on gene ontology (GO)^16^. Specifically, the biological meaning of a gene is given by the GO-terms this gene is associated with as provided by GO. Similarly, the biological meaning of a set of genes is provided by the union of the sets of GO-terms of the individual genes.

## Results

Our analysis is structured into three main parts. In the first part, we study characteristics of the 48 BM signatures individually and comparatively. In the second and third part, we study prognostic prediction capabilities of random gene sets, systematically constructed with two different procedures.

### Biomarker set sizes and GO-term in signatures

**Figure 2:**
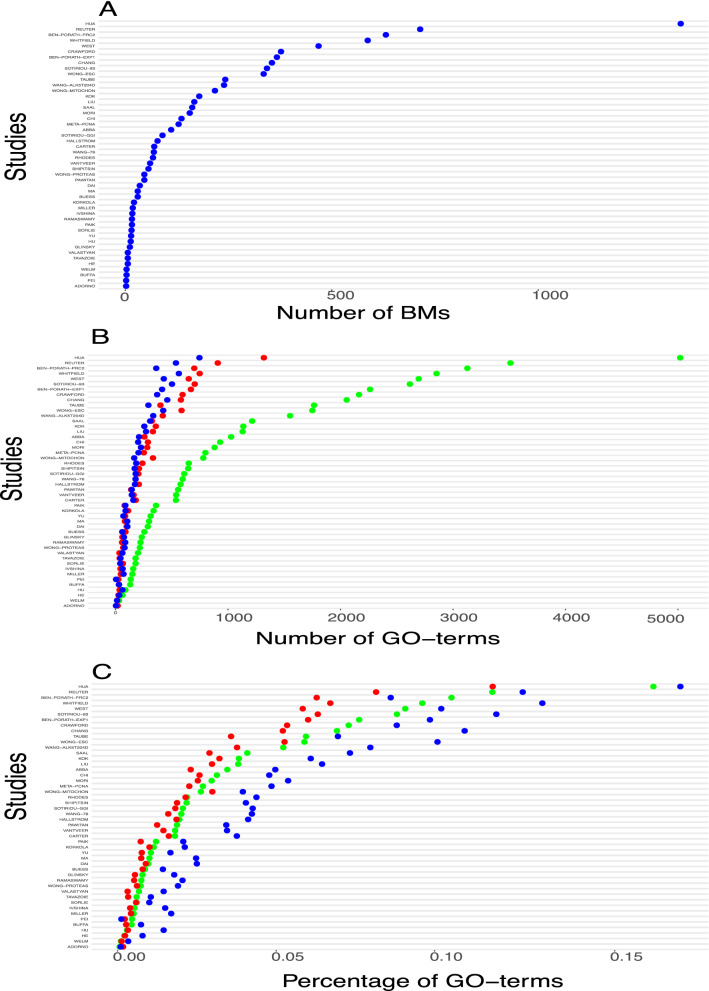
(**A**) Overview of the total number of biomarker genes in each study. (**B**) Shown is the number of GO-terms in each study. The green points correspond to BP, the red points to MF and blue points to CC. (**C**) The percentage of GO-terms of BP, MF and CC used by each study. The color is the same as for B.

In Fig. 2A, we show an overview of the total number of genes in each signature. The name of the signatures are on the y-axis and the x-axis provides information about the size of the BM signatures.

From this figure, one can see that the signature by Adorno and Pei contains the least number of genes (2) whereas Hua has the largest number (1345 genes). That means the size of the signatures varies considerably among the studies and the average size of a signature is 168.9 genes.

**Figure 3:**
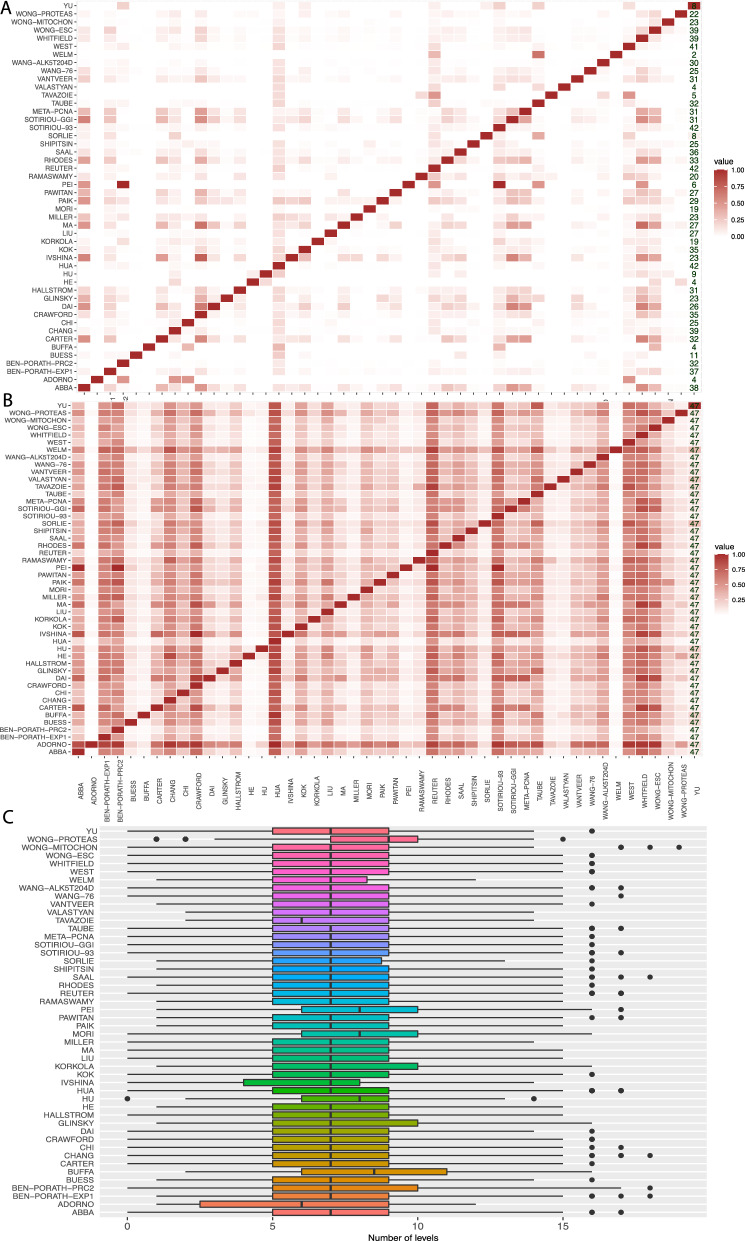
(**A**) Pairwise overlap of genes in BM signatures. (**B**) A: Pairwise overlap of GO-terms in BM signatures. (**C**) The distribution of GO-terms of BP hierarchy levels for each study.

In Fig. 2B and C, we show information about associated GO-terms with the genes in the signatures for the categories: Biological process (BP), molecular function (MF), and cellular component (CC). Currently, there are in total 29, 699 GO-terms from BP, 4202 GO-terms from CC and 11, 148 GO-terms from MF. In Fig. 2B, we show the absolute number of GO-terms in each study with respect to BP (green), MF (red) and CC (blue) whereas Figure 2C shows the corresponding percentage with respect to the total number of GO-terms for each category (i.e., BP, MF and CC).

Overall, from Fig. 2 B one can see that the present GO-terms in the signatures is considerably different from each other. This variation is particularly large for GO-terms of BP (green). Interestingly, if one considers the percentage of present GO-terms (see Fig. 2C) then the differences between the three GO categories (i.e., BP, MF and CC) become much smaller, although, also on this scale the differences between studies are considerable. The average number of GO-terms is 996.7 for BP, 277.9 for MF and 204 for CC and the average percentage is 0.034 for BP, 0.025 for MF and 0.049 for CC.

Using a Spearman rank correlation test, we investigate if the order of the size of biomarker sets (see Fig. 2A) is conserved by the number of GO-terms (see Fig. 2B). As a result, we find p-values of $1.311469e-28$ for BP (green), $3.44238e-35$ for MF (red) and $2.96905e-29$ for CC (blue). Due to the fact that the percentage of GO-terms shown in Fig. 2C has the same order as for the number of GO-terms in Fig. 2B, a comparison of these results leads to the exact same p-values. Overall, the above p-values indicate that the order of all comparisons is highly statistically significant for any sensible significance level $\alpha$. Therefore, the ranking of the biomarker sets with respect to their size is similar to the ranking according to their number of GO-terms, which implies that larger BM signatures contain more GO-terms.

### Pairwise similarity of signatures

For our next analysis, we perform a pairwise comparison of the BM signatures. That means, we study the overlap of common genes and GO-terms among different signatures. In Fig. 3A and B, the results from these pairwise comparisons are shown in form of heat maps. Formally, we define the overlap as follows. Let $S_i$ and $S_j$ be two signature sets consisting either of genes or GO-terms corresponding to these genes. Then we find the percentage $z_i$ of common elements in $S_i$ that are also present in $S_j$ by

2 $$\begin{aligned} x_i&= S_i \cap S_j \end{aligned}$$

3 $$\begin{aligned} z_i&= \frac{|x_i|}{|S_i|}. \end{aligned}$$

Here $z_i$ can assume values between zero and one. We would like to remark that the way we find the overlap is asymmetric, i.e., $z_i \not = z_i$ if $|S_i| \not =|S_j|$. That means the percentage overlap is taken with respect to the first signature set $S_i$.

From comparing the gene overlap (see Fig. 3A), the signature of Pei is the only one that is completely included in two other signatures namely Ben-porath-prc2 and Sotiriou-93. Interestingly, there is no unique signature, which means that each signature has some overlap with at least one other signature. The signature with the least commonality with other signatures is from Welm, which has only genes in common with the signatures of Taube and Reuter. Also the signature from Adorno has only a gene overlap with 4 other signatures. The signatures with the largest number of overlaps are Hua, Reuter and Sotiriou-93. These three signatures are sharing genes with 42 other signatures. This means that the overlap with other signatures varies considerably from 2 to 42. These numbers are added to Fig. 3A in the last column of the heat map.

In contrast to this, the overlap of GO-terms among signatures is shown in Fig. 3B. Also here the overlap among the signatures varies considerably. For instance, the signatures of Hua and Reuter share the highest overlap of 2614 GO-terms, whereas Adorno and He, Adorno and Welm have the lowest overlap of 1 GO-term. However, the most important result is that all signatures share at least some GO-terms with every other signature (see last column). Hence, all signatures have a non-zero overlap in their biological meaning as measured by GO-terms. This is different to the gene-overlap shown in Fig. 3A.

### Hierarchy levels of GO-terms

For our last analysis of the signatures, we are mapping the GO-terms to structural features of a GO-DAG. Specifically, we obtain information about the hierarchy levels of the GO-terms.

In Fig. 3, we show the distributions for the hierarchy levels of the GO-terms of BP. This means that for each signature, the levels of the GO-terms of BP are obtained and a boxplot of the distribution is shown. Interestingly, a large number of signatures exhibit a similar distribution for the levels, and most of the signatures have the same median value of 7 (except for Wong-proteas, Tavazoie, Pei, Mori, Hu, Buffa, Ben-Porath-Prc2 and Adorno). Furthermore, all signatures, besides Wong-proteas, Welm, Tavazoie, Ivshina, Hu, and Glinsky, are symmetric. Specifically, the signatures Wong-proteas, Welm, Ivshina and Hu are skewed to the right whereas the remaining ones are skewed to the left. The Wong-Proteas signature also has the highest median value (9), while Adorno and Tavazoie have the lowest median values (6). The degree of variation for the levels remains virtually the same for most of the signatures with the exception of a few.

These results demonstrate that despite the size differences of the signatures (see Fig. 2A), the differences in the number of GO-terms (see Fig. 2B) and the sparsity in the overlap of genes of the signatures (see Fig. 3A) the biological specificity of the GO-terms is very similar.

### Prognostic prediction capabilities of random gene sets

In the following, we investigate the prognostic prediction capabilities of BM signatures and random gene sets systematically. We start by focusing on BM signatures and random gene sets for which the BM signatures have been removed. Thereafter, we investigate random gene sets for which not only the BM signatures have been removed but also further genes that share common biological processes. This will lead to more stringent insights about the biological meaning of BM signatures.

#### Effect of removing individual BM signatures

The study by^17^ investigated prediction capabilities of random gene sets, $RGS_i$, whereas the genes in $RGS_i$ were randomly sampled from the set $G_i' = G \setminus BM_i$. Here *G* corresponds to the total number of genes in our breast cancer data set and $BM_i$ is the BM signature of study *i*, for $i \in \{1, \dots , 48\}$. The number of genes sampled per random signature is the same as in $BM_i$, i.e., $|RGS_i|=|BM_i|$. We repeat this sampling 1000 times for each study, i.e., we studied 48, 000 random gene sets that have been constructed in this way.

We would like to remark, that the study by^17^ did not apply a multiple testing correction to the obtained p-values despite the fact that multiple hypotheses had been tested. In order to see if these previous results are statistically robust, we repeated their analysis using a conservative Bonferroni correction^24^. Therefore, in total, we study 96, 000 random gene sets with and without Bonferroni correction.

**Figure 4:**
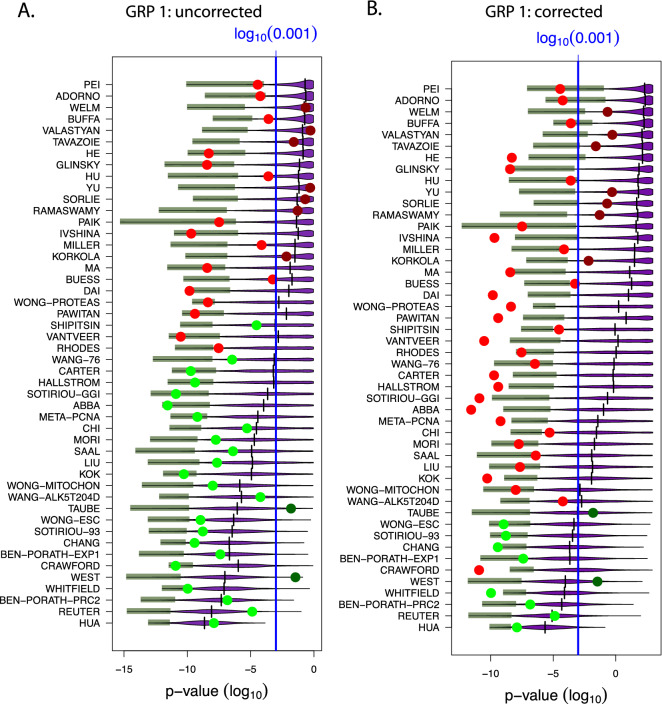
Results for gene removal procedure 1 for the NKI data. Shown are prognostic prediction capabilities of surrogate gene sets for 48 studies after removing BM signatures. Left: Results for uncorrected p-values (as in the original study^17^). Right: Bonferroni corrected p-values.

The results of this analysis are shown in Fig. 4. Here the red/green points are the outcomes of the original BM signatures whereas dark red/dark green colors indicate non-significant values and light colors correspond to significant results. The violet distributions correspond to results from random signatures and the shaded green bars correspond to the lower 3*rd* percentile of these distributions. Furthermore, the horizontal black lines represent the median values of the distribution of random signatures and the long horizontal blue line corresponds to a significance level of $\alpha =0.001$. Note that for the p-values a logarithmic scale (i.e., $log_{10}$) is used.

First, we observe from Fig. 4 that not all BM signatures (big points) lead to significant results. Specifically, the dark red and dark green points correspond to non-significant results whereas the light red and light green points correspond to significant results. This is a result from using different validation data than have been used by the original 48 BM studies. Still, without and with Bonferroni correction there are 39 BM signatures significant in each case. Hence, the remaining 9 signatures do not show prognostic value for independent validation data and lack robustness.

Furthermore, from Fig. 4 without a Bonferroni correction (left), we find that the median p-values of 37 studies are significant (11 studies are not significant) while for a Bonferroni correction (right), we find only 19 significant studies (29 studies are not significant). Also, we find that with and without a Bonferroni correction most lower 3% percentiles (green bars) are significant.

In order to obtain a better understanding of the total number of random gene signatures, we estimate an upper bound of the binomial coefficient ${{n}\atopwithdelims (){k}}$. Here *n* is the available number of genes and *k* is the size of a random biomarker set. The meaning of this binomial coefficient is the total number of random gene sets that can be formed by selecting *k* genes from all available *n* genes.

For our data set the order of magnitude of *n* is $10^4$ and according to Fig. 2A the average size of a BM signature is $k= 10^2$. For the following estimate, only the order of magnitude of *n* and *k* are important as we will see below. Due to the fact that ${{10,000}\atopwithdelims (){100}}$ cannot be evaluated numerically, we estimate an upper bound of this by

4 $$\begin{aligned} {{n}\atopwithdelims (){k}} \le \Big ( \frac{n \cdot e}{k} \Big )^k. \end{aligned}$$

Here *e* is Euler’s number. The right-hand-side of Eq. 4 can be simplified by

5 $$\begin{aligned}&\Big ( \frac{n \cdot e}{k} \Big )^k = 10^x \Rightarrow \end{aligned}$$

6 $$\begin{aligned}{}&x = k \cdot \log _{10} \Big ( \frac{n \cdot e}{k} \Big ) \end{aligned}$$

to obtain the order of magnitude as an exponent of 10. Overall, this leads to the following approximation of the binomial coefficient

7 $$\begin{aligned} {{n}\atopwithdelims (){k}} \le \Big ( \frac{n \cdot e}{k} \Big )^k = 10^{x} = 10^{243} \end{aligned}$$

for values of $n=10,000$ and $k=100$ and *x* given in Eq. 6 (after rounding to integer numbers).

This demonstrates that the average number of random gene sets is in the order of $10^{243}$ and that one percentile of these correspond to $10^{241}$ different random gene sets, for each study. Hence, even for studies for which only about three percent of all random gene sets are significant, corresponding to the lower 3*rd* percentile (green bars) in Fig. 4, the number of such gene sets is very large. In order to distinguish such significant random gene sets from non-significant gene sets we call the former *surrogate gene sets* because they have the same prognostic prediction capabilities as the BM signatures. Hence, the lower 3*rd* percentile corresponds to $10^{241}$ surrogate gene sets.

#### Effect of removing related biological processes

In our next analysis, we go one step further. Instead of only removing BM signatures, we remove also genes that belong to the same biological processes as the genes in the BM signatures (see GRP 2 in the Methods section). Due to the fact that according to the gene ontology (GO) database the biological processes are hierarchically organized, we approach this analysis iteratively by removing successively genes of biological processes on the same hierarchy level. Details of gene removal procedure 2 are described in the Methods section.

**Table 1:** Results for GRP 2 (NKI data) for three signatures. Top: Pei. Middle: Chang. Bottom: Wong–Mitochon. The p-values have been Bonferroni corrected.

Hierachy level	Genes removed	Cum. sum of genes removed	Genes left	GO-terms removed	Cum. sum of GO-terms removed	Acc. (%)	Sig.Acc. (%)
16	3	3	12,135	1	1	3.3	2.5
15	8	11	12,127	3	4	4.0	
14	2	13	12,125	1	5	2.9	
13	177	190	11,948	2	7	4.7	
12	1318	1508	10,630	9	16	3.3	
11	616	2124	10,014	8	24	3.7	
10	432	2556	9582	10	34	3.2	
9	229	2785	9353	11	45	3.4	
8	190	2975	9163	13	58	3.1	
7	197	3172	8966	9	67	2.8	
6	533	3705	8433	24	91	2.5	
5	854	4559	7579	16	107	3.2	
4	837	5396	6742	4	111	4.1	
3	130	5526	6612	4	115	2.5	
2	250	5776	6362	3	118	3.0	
1	56	5832	6306	1	119	3.2	
18	17	17	11,822	1	1	87.0	88.5
17	11	28	11,811	3	4	87.9	
16	3	31	11,808	2	6	89.6	
15	89	120	11,719	8	14	89.2	
14	203	323	11,516	24	38	89.8	
13	819	1142	10,697	41	79	87.4	
12	1932	3074	8765	91	170	87.1	
11	1246	4320	7519	112	282	84.8	
10	1216	5536	6303	153	435	84.3	
9	1326	6862	4977	174	609	81.9	
8	1252	8114	3725	183	792	79.6	
7	1258	9372	2467	193	985	70.8	
6	711	10,083	1756	165	1150	81.2	
5	573	10,656	1183	97	1247	73.2	
4	336	10,992	847	54	1301	88.1	
3	122	11,114	725	27	1328	86.3	
18	17	17	11,913	1	1	78.9	78.0
17	9	26	11,904	3	4	74.9	
14	63	89	11,841	9	13	76.7	
13	228	317	11,613	12	25	78.0	
12	1228	1545	10,385	25	50	78.7	
11	826	2371	9559	53	103	76.3	
10	1054	3425	8505	60	163	75.5	
9	1032	4457	7473	70	233	76.8	
8	949	5406	6524	77	310	78.6	
7	1754	7160	4770	89	399	69.7	
6	810	7970	3960	78	477	68.6	
5	868	8838	3092	67	544	63.4	
4	461	9299	2631	35	579	63.6	
3	339	9638	2292	24	603	65.6	
2	238	9876	2054	12	615	56.0	
1	30	9906	2024	3	618	56.6	

We assess the prediction results again by the p-values from the survival analysis. In addition, we assess the accuracy of predictions by declaring significant p-values as true positives (TPs) and non-significant results as false negatives (FNs). This allows the estimation of accuracy values. These evaluations are obtained for each hierarchy level.

**Figure 5:**
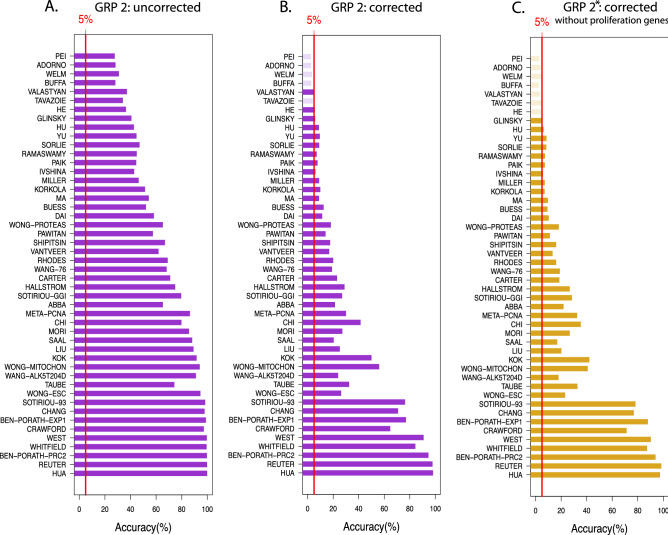
Results for gene removal procedure 2 for the NKI data. (**A**), (**B**) Show results for the minimal accuracy values across all hierarchy levels, whereas A is for uncorrected p-values and B for Bonferroni corrected p-values. (**C**) Results for GRP 2* for removing all GO-terms on all hierarchy levels (Bonferroni corrected).

In Table 1, we show three representative results for the signatures of Pei (top), Chang (middle) and Wong-Mitochon (bottom). The underlying p-values have been Bonferroni corrected. The remaining results for the remaining signatures can be found in the Tables 1 to 96 in the supplementary file. The first column shows the hierarchy level up to which the GO-terms have been removed (see GRP 2 Methods section) and columns two to six give further details about the involved genes and GO-terms. The accuracy (Acc) summarizes the results of 1000 repeats and the overall significant p-values. Finally, Sig. Acc. gives the accuracy when only the signature is removed.

As one can see the accuracy values can be very low (Pei: top) or very high (Chang:middle ) regardless of the hierarchy level, or they can decline toward higher hierarchy levels (Wong–Mitochon: bottom). However, despite this complicated behavior a commonality among all 48 studies is that there is always a non-vanishing percentage of random gene sets that make the correct predictions. Hence, the number of surrogate gene sets is non-zero for all signatures.

This is summarized in Fig. 5. Specifically, the shown accuracy values correspond to the minimal values for each study across all hierarchy levels. For instance, for Chang the minimal accuracy is 70.8% obtained for hierarchy level 7; see Table 1. From this figure one can see that also the resulting minimal accuracy values vary considerably across the studies, however, only 5 studies have values slightly smaller than 5%. All other studies have larger values than 5% and some are even larger than 80%, even for Bonferroni corrected p-values. Examples for the latter are the signatures from West, Whitfield, Ben-Porath-Prc2, Reuter and Hau.

**Figure 6:**
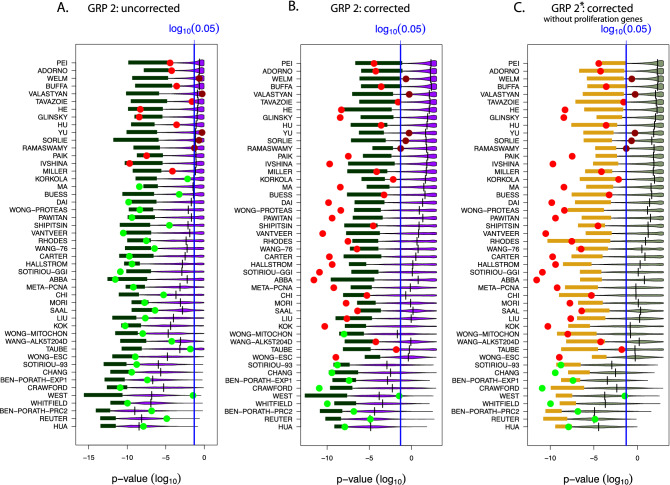
Results for gene removal procedure 2 for the NKI data. The results in (**A**) and (**B**) are for the minimal accuracy (see Fig. 5A,B), whereas A is for uncorrected p-values and B for Bonferroni corrected p-values. The results in (**C**) correspond to Fig. 5C where all GO-terms on all hierarchy levels have been removed and, in addition, all proliferation genes have been removed.

For each hierarchy level of each study, one can investigate the resulting distribution of p-values for the random gene sets (similar to Fig. 4). Due to the fact that for each study many hierarchy levels have been studied (see Table 1 or the supplementary Tables 1 to 96) there are more than 1000 such distributions for all studies. For instance, for Pei there are 16 such distributions corresponding to 16 hierarchy levels (see Table 1). In order to simplify the presentation, we show only results for the minimal accuracy values in Fig. 5. The corresponding results are shown in Fig. 6. Interestingly, these results are qualitatively comparable to the results shown in Fig. 4. However, quantitatively, the difference is that *in average* these p-values are slightly larger. This implies, e.g., that the median values of less studies are significant. Specifically, in Fig. 6 the median values of 33 (without Bonferroni correction) respectively 10 (with Bonferroni correction) studies are significant.

In order to estimate the number of surrogate gene sets, we perform a similar approximation of the binomial coefficient as in Eq. 7, however, considering the reduced number of available genes. From the tables in the Supplementary File we observe, in average, $n=1000$. Considering this, we obtain $x=143$. Therefore, the total number of random gene sets constructed with GRP 2 is

8 $$\begin{aligned} {{n}\atopwithdelims (){k}} \le 10^{143} . \end{aligned}$$

Also this number is very large but a factor of $10^{100}$ smaller than the number of random gene sets obtained in Eq. 7.

From Fig. 5 and 6 (here the 3rd percentiles are highlighted in green) one can see that also for this procedure a certain percentile or random gene sets lead to the correct prediction outcome. Hence, the number of surrogate gene sets is for GRP 2 in the order of $10^{141}$.

**Figure 7:**
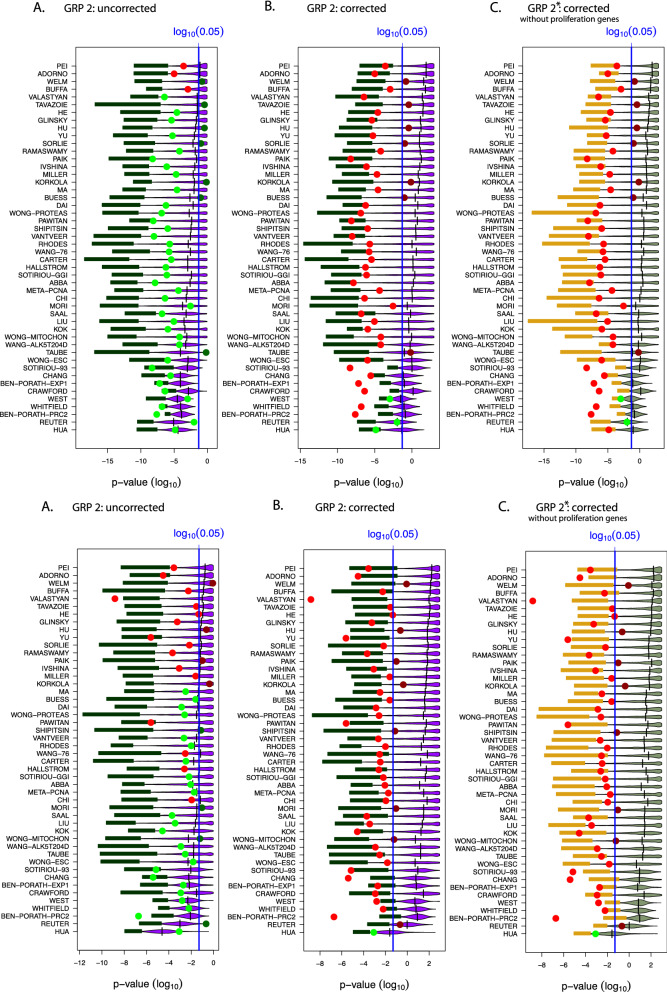
Results for gene removal procedure 2 and the SWE data similar to Fig. 6. Top row: Patient samples contain LumA, LumB, Basal and Her2. Bottom row: Patient samples contain LumA and Her2.

**Figure 8:**
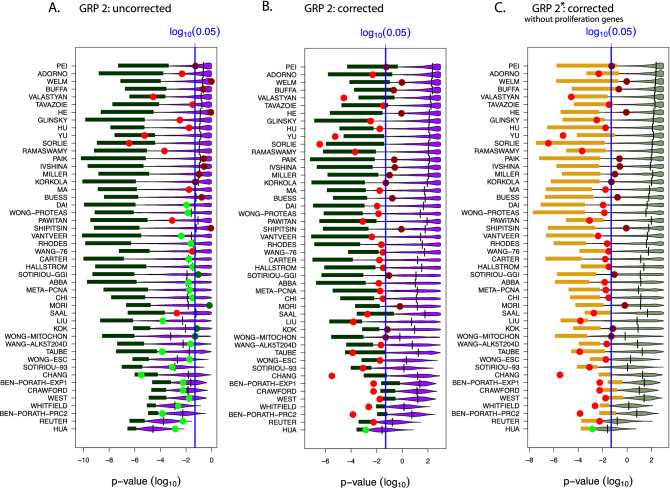
Results for gene removal procedure 2 and SWE data similar to Fig. 6. Patient samples contain LumA and LumB.

Finally, we repeat the above analysis for another data set from^18^, in order to demonstrate the robustness of our results. In Figs. 7 and 8 we show results for the SWE data. Specifically, in Fig. 7 (top row) we use patient samples for LumA, LumB and Her2, in Fig. 7 (bottom row) LumA and Her2 and in Fig. 8 LumA and LumB. As one can see our results for the NKI data are confirmed for the SWE data for different subtypes of cancer. Other combinations of the subtypes give similar results (not shown).

## Discussion

In this paper, we conducted a systematic study investigating the prognostic prediction capabilities of random gene sets. For this, we defined two different gene removal procedures (GRP 1 and GRP 2) for a constrained-sampling of random gene sets.

For clarity, we distinguish in our paper between three different types of genes set. The first one, called a signature, is a gene set identified in a targeted way. Typically, such genes are identified because it is assumed that they are biologically informative for a particular problem. In addition, if used in a prognostic prediction task such a signature yields statistically significant results, which evidences practically that the signature is indicative for the disease progression of patients. In contrast, a random gene set is obtained by randomly sampling genes from an available gene pool. No particular meaning or role is attributed to such genes before sampling. Lastly, a surrogate gene set is a random gene set that has the same prognostic prediction capabilities as a signature. In our case this is indicated by a significant p-value from a survival analysis.

### Results from gene removal procedure 1

The results from GRP 1 are summarized in Fig. 4. From this figure, one obtains the following interpretations.

*Most random signatures are significantly associated with prognostic outcome:* This is only correct for random signatures with a median value that is statistically significant, because the median corresponds to 50% of the population. Hence, a significant median indicates that 50% of the surrogate signatures are significant. In summary, without a Bonferroni correction this is correct for 37 (77.1%) studies and with Bonferroni correction for 19 (39.6%) studies (see Fig. 4). Hence, this statement is signature-dependent and does not hold generally.

*Many surrogate signatures are significantly associated with prognostic outcome:* This statement is correct for surrogate signatures for which a certain percentile of the surrogate signatures is statistically significant. From Fig. 4 one can see that this is correct for the lower 3rd percentiles for all studies, with and without a Bonferroni correction.

*The number of surrogate signatures which are significantly associated with prognostic outcome is very large:* Despite the fact that not all median values for all signatures are significant, the number of surrogate signatures that are significant is for each study very lage. This result has been obtained from approximating the upper bound of the binomial coefficient ${{n}\atopwithdelims (){k}}$ where *n* is the number of available genes and *k* is the size of a surrogate gene set. As an approximation we found

9 $$\begin{aligned} {{n}\atopwithdelims (){k}} \le 10^{243} \end{aligned}$$

for values of $n=10,000$ and $k=100$.

### Results from gene removal procedure 2

The results from GRP 2 are an extension of GRP 1 in the sense that the genes available for random sampling are further constricted. That means instead of only removing BM signatures, in addition, also genes related to the same biological processes are removed. Hence, the random gene sets obtained from this procedure are less biologically similar to the BM signatures.

The initial motivation for exploring GRP 2 came from the observation that the overlap of biological processes present in random gene sets and in BM signatures is non-zero. That means whenever at least one gene in a random gene set belongs to a GO-term that is also present for a BM signature, possibly for a different gene, the random gene set and the BM signature have this GO-term in common. Numerically, we find the average number of common GO-terms (corresponding to BP) across all signatures is 341. We find the largest overlap for Hua with 2602 and the smallest for Adorno with 1 GO-term. These numbers are understandable because Hua contains the largest number of GO-terms (over 5000) whereas Adorno contains the smallest number (19 GO-terms of BP); see Fig. 2.

The results from GRP 2 for the NKI data are summarized in Figs. 5 and 6 (and for the SWE data in Figs. 7 and 8). It is interesting to note that the qualitative results are similar for GRP 2 and GRP 1. That means even by removing genes related to the same biological processes as the signature genes, the prognostic prediction capabilities of surrogate gene sets can be confirmed. Importantly, qualitatively, GRP 1 and GRP 2 are entirely different with respect to their biological meaning. Specifically, we designed GRP 2 in a way that the procedure allows the gradual removal of more and more *biological meaning* from random gene sets. This is accomplished by a ranking of GO-terms according to their hierarchy levels because it is know that GO-terms in a GO-DAG on higher levels contain biological information that is more specific than GO-terms on lower levels^25^. Due to the fact that GRP 2 removes genes, associated with certain GO-terms, gradually from high to low hierarchy levels, we were able to study this effect explicitly; see Table 1 and Tables 1 to 96 in the Supplementary File (for the NKI data). We would like to remark that removal of GO-terms from all hierarchy levels (corresponding to the last step of GRP 2) results in random gene sets with no biological similarity to the original BM signature. Hence, per construction, such random gene sets have a biological similarity of zero with the original BM signature.

Considering the biological differences in the meaning of random genes sets resulting from GRP 1 and GRP 2 the results in Figs. 5 and 6 are remarkable because it means that any biological justification given for the selection of an original BM signature is anecdotal. Specifically, by removing genes related to the same biological processes as the signature genes we eliminate the possibility of *accidentally* selecting genes for a random gene set that share the same biological interpretation as the original BM signature. Hence, any biological interpretation of such a BM signature is meaningless because we demonstrated that one can find surrogate gene sets with the same prediction capability but entirely different biological interpretations due to zero overlap in the GO-terms of involved genes. This is also true for GRP 2* where additionally proliferation genes have been removed (see Fig. 6C).

We would like to remark that the study by^17^ did not allow this conclusion because BM signatures have not been removed nor genes from associated biological processes. This leaves the possibility of *accidentally* selecting genes for a random gene set that share the same biological interpretation as the original BM signatures because these genes belong to the same biological processes as indicated by common GO-terms in the domain BP.

Our study is also different to^21^ where the investigation by^17^ has been extended by removing proliferation genes. The problem with their design is that resulting random gene sets can still have a non-vanishing overlap of common GO-terms and, hence, share to a certain extend biological meaning with a signature. Instead, we aimed at the elimination of all common GO-terms so that the resulting random gene sets have a different biological meaning. Further constraining of GRP 2 by additionally removing proliferation genes, as studied in^21^, which we named GRP 2*, does not change our main result.

Taking a more specific look into some of the studies we used for our analysis allows to make this point more clear. For instance, the study by^26^ identified a BM signature by computationally investigating 42 breast cancer gene expression studies. After demonstrating the prognostic capability of their signature the biological importance of these genes has been discussed and their functional role has been characterized as cell cycle process related and response to steroid hormone stimulus. Similarly, in the studies by Carter^27^, Chi^28^, Saal^29^, Shipitsin^30^ and West^31^ the biological importance of their signatures pointed to chromosomal instability, hypoxia response, PI3K pathway signaling, TGF-β signaling pathway and stromal response respectively. However, based on our results, none of these biological interpretations established a causal explanation of the underlying cancer biology because one can always find alternative gene sets, which we called surrogate gene sets, that contain neither genes from their signatures nor from genes with related biological processes (nor from proliferation genes) but achieve the same prognostic predictions.

From these and other studies, one can derive the following general pattern that can be found in many prognostic breast cancer studies. First, signature genes are identified by computational, experimental or mixed-approaches and, second, the biological relevance of the signature genes is discussed. Our results demonstrate that neither step is necessary. The first step can be omitted because we showed that a constrained random sampling can lead to surrogate gene sets with the same prognostic prediction capabilities. Hence, any sophisticated, e.g., biology-driven selection process is equivalent to a random selection process. From our analysis we found that the probability that such a random gene set is actually a surrogate gene set is in the percentage range.

The second step can be omitted because we showed that by GRP 2 one can systematically construct surrogate gene sets with an entirely different biological meaning as the signature genes. Specifically, due to the fact that we remove systematically all genes related to any biological process of the signature genes, none of the genes in a surrogate gene set can belong to any of these biological processes. Formally, this can be written as follows (for the removal of all hierarchy levels). For any signature

10 $$\begin{aligned} BM = \{g_1, \dots , g_m\} \end{aligned}$$

consisting of *m* genes and corresponding GO-term set

11 $$\begin{aligned} GT = \{GO_1, \dots , GO_t\} \end{aligned}$$

representing all GO-terms of the genes in *BM* and any surrogate gene set

12 $$\begin{aligned} SGS = \{g'_1, \dots , g'_m\} \end{aligned}$$

consisting of *m* genes and corresponding GO-term set

13 $$\begin{aligned} GT' = \{GO'_1, \dots , GO'_{t'}\} \end{aligned}$$

representing all GO-terms of the genes in *SGS*, with *t* possibly different to $t'$, the two sets *GT* and $GT'$ are disjoint, i.e.,

14 $$\begin{aligned} GT \cap GT' = \emptyset . \end{aligned}$$

For our results shown in the Tables 1 to 144 (Supplementary File), including also the removal of proliferation genes, this holds for the last row in these tables, i.e., the highest level. Hence, due to the fact that the signature genes (i.e., *BM*) and the surrogate gene set (i.e., *SGS*) do not share any GO-term they have a complementary biological meaning. Furthermore, there is not just one surrogate gene set but in the order of $10^{141}$ different sets. This demonstrates that the biological discussion of *BM* is meaningless because one can find a huge number of surrogate gene sets with a plurality of biological meanings.

In contrast to studies investigating the problem of reproducibility of biomedical results^32^ requiring the adjustment of approaches, our paper is different because our results point to a fundamental lack of a commonly used framework which is unfixable. As a generalization of our results for 48 signatures, we assert that a signature with a sensible biological interpretation cannot be found within the studied prognostic framework utilizing survival analysis. More formally, this means the commonly used prognostic framework is no causal model^33^.

In conclusion, we demonstrated that the common assumption that “A reliable set of predictive genes also will contribute to a better understanding of the biological mechanism of metastasis”^7^ is not true.

### Falsification mechanism to test biological meaning of prognostic signatures

For testing the validity of general signatures, we suggest the following procedure to test if it is justified to investigate the biological meaning of a prognostic signature of breast cancer.

1. *G* : total number of genes in a breast cancer dataset.
*Optional step: Removing proliferation genes in *PG* from *G*. The set *PG* contains proliferation genes. This gives a new set of genes $G^*$ with $G^*$ = $G \setminus PG$.
3. $BM: \{g_1,\ldots ,g_m\}$. *BM* is the gene signature and $g_1,\ldots ,g_m$ are the genes in the corresponding signature.
4. Mapping of the genes in *BM* to GO-terms. This gives:
  15 $$\begin{aligned} BM = \{g_1,\ldots ,g_m\} \rightarrow \{GO_1,\ldots ,GO_t\} . \end{aligned}$$
  Note, each gene can be connected to more than one GO-term. For this reason $m \le t$.
5. Mapping of the GO-terms to genes. This gives:
  16 $$\begin{aligned} GO_i \rightarrow g(i) = \{g_1(i), \ldots ,g_k(i)\} . \end{aligned}$$
  for all GO-terms *i* with $i \in \{1, \dots , t\}$.
6. Delete all the genes in $D = \cup _{i \in \{1, \dots , t\}} g(i)$ from *G*. This results in a new gene set given by $G'$= $G \setminus D$.
7. From $G'$, sample new sets of random genes of size |*BM*| and perform the prognostic task. This is repeated 1000 times.
8. Application of a Bonferroni correction to the p-values and assessing the performance for a significance level of $\alpha$.

From numerical analyses, we found that 1000 repeats are sufficient to estimate the tail distribution of random gene sets because, for the signatures studied in this paper, the *probability to be a surrogate gene set* ($p_{sgs}$) is in average 3% percent or higher. However, other signatures may require larger repeats due to the reciprocal relation between these entities, i.e., $\#\text{ repeats } > 1/p_{sgs}$.

If this procedure does not result in any surrogate gene set with the same prognostic prediction capabilities, the BM signature has a biological meaning that deserves to be discussed. Otherwise the BM signature has no sensible biological interpretation, which is the case for the 48 signatures studied in this paper.

## Conclusion

In this paper, we shed light on the biological interpretability of BM signatures for the prognostic prediction of breast cancer. Our results demonstrate that none of the 48 studied signatures has a sensible biological interpretation because for each, surrogate gene sets can be found that perform the same task, however, belonging to different biological processes. This implies that every signature (random or not) can just serve as a *black-box* prediction model without a biological interpretation. We believe that this has wider implications, even beyond biomedicine, to general machine learning and artificial intelligence models but this remains to be studied^34^. In addition, we proposed a procedure to test the biological meaning of prognostic signatures of breast cancer. This test could avoid further confusion in the literature about the biological meaning of prognostic signatures.

It is widely know that prognostic signatures of breast cancer are very heterogeneous and sensitive to changes in the studied perspective. For this reason, we assumed in this paper a higher conceptual ground, based on a systems-view, in order to study a common aspect shared by many signatures that allows to pierce through the unavoidable variability and heterogeneity. This concept goes back to the roots of systems biology as envisioned in^35,36^.

## Supplementary Information

Supplementary Information.
