# Supplementary material for: Prognostic gene expression signatures of breast cancer are lacking a sensible biological meaning
Source: Sci Rep. 2021 Jan 8;11:156. doi: 10.1038/s41598-020-79375-y (PMC7794581; doi:10.1038/s41598-020-79375-y)
Supplement: Supplementary file 1 — Supplementary Information. [file 41598_2020_79375_MOESM1_ESM.pdf]

Supplementary information: Prognostic gene expression signatures of breast cancer are lacking a sensible biological meaning

Kalifa Manjang, Shailesh Tripathi, Olli Yli-Harja, Matthias Dehmer, Galina Glazko, Frank Emmert-Streib

November 24, 2020

Contents

1 Additional results **5**

2 GRP 2 without Bonferroni correction **6**

2.1 ABBA 6
2.2 ADORNO 6
2.3 BEN-PORATH-EXP1 7
2.4 BEN-PORATH-PRC2 7
2.5 BUESS 8
2.6 BUFFA 8
2.7 CARTER 9
2.8 CHANG 9
2.9 CHI 10
2.10 CRAWFORD 10
2.11 DAI 11
2.12 GLINSKY 11
2.13 HALLSTROM 12
2.14 HE 12
2.15 HU 13
2.16 HUA 13
2.17 IVSHINA 14
2.18 KOK 14
2.19 KORKOLA 15
2.20 LIU 15
2.21 MA 16
2.22 MILLER 16
2.23 MORI 17
2.24 PAIK 17
2.25 PAWITAN 18
2.26 PEI 18
2.27 RAMASWAMY 19

2.28 REUTER 19
2.29 RHODES 20
2.30 SAAL 20
2.31 SHIPITSIN 21
2.32 SORLIE 21
2.33 SOTIRIOU-93 22
2.34 SOTIRIOU-GGI 22
2.35 META-PCNA 23
2.36 TAUBE 23
2.37 TAVAZOIE 24
2.38 VALASTYAN 24
2.39 VANTVEER 25
2.40 WANG-76 25
2.41 WANG-ALK5T204D 26
2.42 WELM 26
2.43 WEST 27
2.44 WHITFIELD 27
2.45 WONG-ESC 28
2.46 WONG-MITOCHON 28
2.47 WONG-PROTEAS 29
2.48 YU 29

3 GRP 2 with Bonferroni correction 30
3.1 ABBA 30
3.2 ADORNO 30
3.3 BEN-PORATH-EXP1 31
3.4 BEN-PORATH-PRC2 31
3.5 BUESS 32
3.6 BUFFA 32
3.7 CARTER 33
3.8 CHANG 33
3.9 CHI 34
3.10 CRAWFORD 34
3.11 DAI 35
3.12 GLINSKY 35
3.13 HALLSTROM 36
3.14 HE 36
3.15 HU 37
3.16 HUA 37
3.17 IVSHINA 38
3.18 KOK 38
3.19 KORKOLA 39
3.20 LIU 39
3.21 MA 40
3.22 MILLER 40
3.23 MORI 41
3.24 PAIK 41
3.25 PAWITAN 42
3.26 PEI 42
3.27 RAMASWAMY 43

3.28 REUTER . . . 43
3.29 RHODES . . . 44
3.30 SAAL . . . 44
3.31 SHIPITSIN . . . 45
3.32 SORLIE . . . 45
3.33 SOTIRIOU-93 . . . 46
3.34 SOTIRIOU-GGI . . . 46
3.35 META-PCNA . . . 47
3.36 TAUBE . . . 47
3.37 TAVAZOIE . . . 48
3.38 VALASTYAN . . . 48
3.39 VANTVEER . . . 49
3.40 WANG-76 . . . 49
3.41 WANG-ALK5T204D . . . 50
3.42 WELM . . . 50
3.43 WEST . . . 51
3.44 WHITFIELD . . . 51
3.45 WONG-ESC . . . 52
3.46 WONG-MITOCHON . . . 52
3.47 WONG-PROTEAS . . . 53
3.48 YU . . . 53

4 GRP 2* with Bonferroni correction and removal of proliferation genes 54

4.1 ABBA . . . 54
4.2 ADORNO . . . 54
4.3 BEN-PORATH-EXP1 . . . 55
4.4 BEN-PORATH-PRC2 . . . 55
4.5 BUESS . . . 56
4.6 BUFFA . . . 56
4.7 CARTER . . . 57
4.8 CHANG . . . 57
4.9 CHI . . . 58
4.10 CRAWFORD . . . 58
4.11 DAI . . . 59
4.12 GLINSKY . . . 59
4.13 HALLSTROM . . . 60
4.14 HE . . . 60
4.15 HU . . . 61
4.16 HUA . . . 61
4.17 IVSHINA . . . 62
4.18 KOK . . . 62
4.19 KORKOLA . . . 63
4.20 LIU . . . 63
4.21 MA . . . 64
4.22 MILLER . . . 64
4.23 MORI . . . 65
4.24 PAIK . . . 65
4.25 PAWITAN . . . 66
4.26 PEI . . . 66

4.27 RAMASWAMY . . . 67
4.28 REUTER . . . 67
4.29 RHODES . . . 68
4.30 SAAL . . . 68
4.31 SHIPITSIN . . . 69
4.32 SORLIE . . . 69
4.33 SOTIRIOU-93 . . . 70
4.34 SOTIRIOU-GGI . . . 70
4.35 META-PCNA . . . 71
4.36 TAUBE . . . 71
4.37 TAVAZOIE . . . 72
4.38 VALASTYAN . . . 72
4.39 VANTVEER . . . 73
4.40 WANG-76 . . . 73
4.41 WANG-ALK5T204D . . . 74
4.42 WELM . . . 74
4.43 WEST . . . 75
4.44 WHITFIELD . . . 75
4.45 WONG-ESC . . . 76
4.46 WONG-MITOCHON . . . 76
4.47 WONG-PROTEAS . . . 77
4.48 YU . . . 77

5 Overlap of genes and GO-terms 78
5.1 Gene signatures overlap with the 433 genes . . . 79
5.2 Comparison between the signatures . . . 79
5.3 Outcome association for the 433 genes . . . 79

1 Additional results

In this supplementary document, we presented additional results obtained from the outcome association for each of the 48 gene signatures for the NKI data.

2 GRP 2 without Bonferroni correction

Results for GRP 2. The results for the uncorrected p-values are presented. The Sig.acc.(%) column gives the accuracy obtained when only the initial genes in the signature have been omitted. The results in all other columns are for GRP 2.

2.1 ABBA

Hierachy Level	Genes removed	Cum. sum of genes removed	Genes left	GO-terms removed	Cum. sum of GO-terms removed	Acc. (%)	Sig.acc. (%)
16	8	8	12027	2	2	86.3	88.1
15	13	21	12014	5	7	86.0	
14	129	150	11885	12	19	87.1	
13	473	623	11412	19	38	86.1	
12	1808	2431	9604	45	83	86.2	
11	1104	3535	8500	65	148	87.8	
10	927	4462	7573	70	218	87.2	
9	1267	5729	6306	100	318	85.4	
8	948	6677	5358	102	420	85.0	
7	1301	7978	4057	108	528	80.9	
6	655	8633	3402	94	622	82.1	
5	869	9502	2533	67	689	81.2	
4	712	10214	1821	39	728	75.5	
3	260	10474	1561	23	751	73.0	
2	319	10793	1242	16	767	65.3	
1	16	10809	1226	4	771	65.8	

Table 1: ABBA

2.2 ADORNO

Hierachy Level	Genes removed	Cum. sum of genes removed	Genes left	GO-terms removed	Cum. sum of GO-terms removed	Acc. (%)	Sig.acc. (%)
12	697	697	11441	2	2	29.5	29.8
11	277	974	11164	2	4	30.3	
10	615	1589	10549	1	5	29.9	
8	18	1607	10531	2	7	29.4	
7	464	2071	10067	1	8	32.6	
6	101	2172	9966	3	11	28.3	
4	46	2218	9920	1	12	29.8	
3	375	2593	9545	1	13	30.1	
2	363	2956	9182	3	16	28.5	
1	107	3063	9075	2	18	30.2	

Table 2: ADORNO

2.3 BEN-PORATH-EXP1

Hierachy Level	Genes removed	Cum. sum of genes removed	Genes left	GO-terms removed	Cum. sum of GO-terms removed	Acc. (%)	Sig.acc. (%)
18	17	17	11796	1	1	98.9	99.6
17	1	18	11795	1	2	99.3	
16	39	57	11756	3	5	99.4	
15	91	148	11665	13	18	99.6	
14	301	449	11364	29	47	99.5	
13	861	1310	10503	55	102	99.4	
12	1854	3164	8649	95	197	98.9	
11	1125	4289	7524	121	318	99.4	
10	1286	5575	6238	177	495	99.2	
9	1574	7149	4664	197	692	99.4	
8	1093	8242	3571	188	880	99.2	
7	1138	9380	2433	170	1050	99.6	
6	658	10038	1775	151	1201	99.4	
5	605	10643	1170	101	1302	99.6	
4	348	10991	822	46	1348	100.0	

Table 3: BEN-PORATH-EXP1

2.4 BEN-PORATH-PRC2

Hierachy Level	Genes removed	Cum. sum of genes removed	Genes left	GO-terms removed	Cum. sum of GO-terms removed	Acc. (%)	Sig.acc. (%)
17	12	12	11678	4	4	99.9	100.0
16	59	71	11619	5	9	100.0	
15	128	199	11491	20	29	99.9	
14	284	483	11207	39	68	100.0	
13	833	1316	10374	69	137	99.8	
12	1657	2973	8717	110	247	100.0	
11	1002	3975	7715	136	383	99.9	
10	1151	5126	6564	158	541	99.9	
9	1472	6598	5092	222	763	99.8	
8	1136	7734	3956	208	971	99.9	
7	1124	8858	2832	181	1152	99.7	
6	719	9577	2113	179	1331	100.0	
5	577	10154	1536	113	1444	99.7	

Table 4: BEN-PORATH-PRC2

2.5 BUESS

Hierachy Level	Genes removed	Cum. sum of genes removed	Genes left	GO-terms removed	Cum. sum of GO-terms removed	Acc. (%)	Sig.acc. (%)
16	25	25	12093	1	1	60.7	60.2
14	3	28	12090	2	3	58.9	
13	296	324	11794	10	13	59.4	
12	1389	1713	10405	14	27	61.3	
11	606	2319	9799	17	44	60.2	
10	642	2961	9157	17	61	56.5	
9	595	3556	8562	27	88	57.5	
8	315	3871	8247	19	107	58.2	
7	1513	5384	6734	36	143	56.9	
6	590	5974	6144	29	172	54.8	
5	723	6697	5421	24	196	54.2	
4	627	7324	4794	16	212	52.1	
3	246	7570	4548	5	217	52.8	
2	47	7617	4501	3	220	53.0	
1	82	7699	4419	2	222	56.8	

Table 5: BUESS

2.6 BUFFA

Hierachy Level	Genes removed	Cum. sum of genes removed	Genes left	GO-terms removed	Cum. sum of GO-terms removed	Acc. (%)	Sig.acc. (%)
16	24	24	12114	3	3	29.8	31.4
15	26	50	12088	2	5	29.3	
14	29	79	12059	4	9	29.7	
13	143	222	11916	5	14	32.2	
12	1424	1646	10492	10	24	29.1	
11	497	2143	9995	9	33	28.9	
10	212	2355	9783	14	47	31.5	
9	462	2817	9321	10	57	29.9	
8	161	2978	9160	9	66	31.5	
7	544	3522	8616	17	83	28.2	
6	262	3784	8354	11	94	31.0	
5	422	4206	7932	10	104	30.8	
4	1088	5294	6844	7	111	30.0	
3	337	5631	6507	3	114	29.7	
2	1	5632	6506	1	115	33.3	

Table 6: BUFFA

2.7 CARTER

Hierachy Level	Genes removed	Cum. sum of genes removed	Genes left	GO-terms removed	Cum. sum of GO-terms removed	Acc. (%)	Sig.acc. (%)
16	6	6	12065	1	1	79.6	79.6
15	29	35	12036	2	3	77.7	
14	62	97	11974	3	6	80.0	
13	286	383	11688	9	15	79.7	
12	1554	1937	10134	30	45	79.1	
11	983	2920	9151	32	77	78.7	
10	538	3458	8613	34	111	77.7	
9	1368	4826	7245	65	176	78.4	
8	544	5370	6701	54	230	79.1	
7	1178	6548	5523	65	295	76.8	
6	595	7143	4928	51	346	74.8	
5	1068	8211	3860	36	382	75.6	
4	558	8769	3302	26	408	76.4	
3	466	9235	2836	23	431	75.6	
2	322	9557	2514	10	441	72.0	
1	109	9666	2405	4	445	71.0	

Table 7: CARTER

2.8 CHANG

Hierachy Level	Genes removed	Cum. sum of genes removed	Genes left	GO-terms removed	Cum. sum of GO-terms removed	Acc. (%)	Sig.acc. (%)
18	17	17	11822	1	1	99.2	99.6
17	11	28	11811	3	4	99.7	
16	3	31	11808	2	6	99.5	
15	89	120	11719	8	14	99.8	
14	203	323	11516	24	38	99.6	
13	819	1142	10697	41	79	99.5	
12	1932	3074	8765	91	170	99.8	
11	1246	4320	7519	112	282	99.9	
10	1216	5536	6303	153	435	99.0	
9	1326	6862	4977	174	609	99.2	
8	1252	8114	3725	183	792	98.5	
7	1258	9372	2467	193	985	98.7	
6	711	10083	1756	165	1150	99.7	
5	573	10656	1183	97	1247	97.9	
4	336	10992	847	54	1301	100.0	
3	122	11114	725	27	1328	99.9	

Table 8: CHANG

2.9 CHI

Hierachy Level	Genes removed	Cum. sum of genes removed	Genes left	GO-terms removed	Cum. sum of GO-terms removed	Acc. (%)	Sig.acc. (%)
16	20	20	12014	1	1	89.0	90.6
15	56	76	11958	4	5	90.2	
14	191	267	11767	13	18	90.1	
13	553	820	11214	18	36	90.5	
12	1642	2462	9572	35	71	91.0	
11	939	3401	8633	50	121	91.5	
10	1070	4471	7563	65	186	91.6	
9	1077	5548	6486	76	262	92.4	
8	1086	6634	5400	96	358	93.3	
7	1625	8259	3775	99	457	89.9	
6	648	8907	3127	92	549	89.4	
5	821	9728	2306	72	621	87.6	
4	536	10264	1770	49	670	87.9	
3	229	10493	1541	25	695	87.6	
2	255	10748	1286	14	709	81.1	
1	13	10761	1273	3	712	79.8	

Table 9: CHI

2.10 CRAWFORD

Hierachy Level	Genes removed	Cum. sum of genes removed	Genes left	GO-terms removed	Cum. sum of GO-terms removed	Acc. (%)	Sig.acc. (%)
16	26	26	11791	3	3	98.9	98.8
15	90	116	11701	11	14	99.3	
14	218	334	11483	24	38	99.1	
13	925	1259	10558	57	95	98.9	
12	1858	3117	8700	88	183	99.1	
11	1127	4244	7573	125	308	99.3	
10	1281	5525	6292	163	471	98.8	
9	1456	6981	4836	196	667	99.1	
8	1139	8120	3697	190	857	99.3	
7	1120	9240	2577	174	1031	99.1	
6	670	9910	1907	150	1181	99.2	
5	643	10553	1264	99	1280	97.3	
4	376	10929	888	56	1336	99.9	

Table 10: CRAWFORD

2.11 DAI

Hierachy Level	Genes removed	Cum. sum of genes removed	Genes left	GO-terms removed	Cum. sum of GO-terms removed	Acc. (%)	Sig.acc. (%)
16	6	6	12099	1	1	65.3	63.5
14	18	24	12081	1	2	64.6	
13	150	174	11931	5	7	62.7	
12	1388	1562	10543	9	16	64.7	
11	909	2471	9634	23	39	64.9	
10	602	3073	9032	19	58	63.2	
9	980	4053	8052	36	94	66.1	
8	435	4488	7617	25	119	66.5	
7	1137	5625	6480	42	161	65.6	
6	769	6394	5711	34	195	61.8	
5	728	7122	4983	20	215	59.4	
4	1238	8360	3745	15	230	59.2	
3	299	8659	3446	11	241	64.6	
2	432	9091	3014	7	248	59.4	
1	37	9128	2977	2	250	58.3	

Table 11: DAI

2.12 GLINSKY

Hierachy Level	Genes removed	Cum. sum of genes removed	Genes left	GO-terms removed	Cum. sum of GO-terms removed	Acc. (%)	Sig.acc. (%)
16	6	6	12124	1	1	44.4	46.8
15	6	12	12118	2	3	44.4	
14	27	39	12091	2	5	41.5	
13	255	294	11836	6	11	41.5	
12	1503	1797	10333	12	23	45.1	
11	384	2181	9949	17	40	44.4	
10	615	2796	9334	18	58	44.2	
9	658	3454	8676	18	76	42.2	
8	198	3652	8478	16	92	43.0	
7	684	4336	7794	26	118	44.9	
6	513	4849	7281	26	144	41.7	
5	839	5688	6442	21	165	43.9	
4	1162	6850	5280	17	182	43.3	
3	137	6987	5143	5	187	42.4	
2	110	7097	5033	3	190	44.6	
1	26	7123	5007	1	191	40.7	

Table 12: GLINSKY

2.13 HALLSTROM

Hierachy Level	Genes removed	Cum. sum of genes removed	Genes left	GO-terms removed	Cum. sum of GO-terms removed	Acc. (%)	Sig.acc. (%)
15	69	69	12009	4	4	80.1	79.0
14	64	133	11945	4	8	78.7	
13	463	596	11482	13	21	78.8	
12	1547	2143	9935	24	45	77.0	
11	826	2969	9109	32	77	76.2	
10	909	3878	8200	51	128	76.6	
9	1195	5073	7005	54	182	81.7	
8	841	5914	6164	66	248	80.9	
7	1711	7625	4453	76	324	80.1	
6	593	8218	3860	67	391	82.0	
5	1024	9242	2836	48	439	83.1	
4	582	9824	2254	26	465	84.4	
3	220	10044	2034	16	481	81.2	
2	323	10367	1711	9	490	79.8	
1	21	10388	1690	4	494	74.9	

Table 13: HALLSTROM

2.14 HE

Hierachy Level	Genes removed	Cum. sum of genes removed	Genes left	GO-terms removed	Cum. sum of GO-terms removed	Acc. (%)	Sig.acc. (%)
15	6	6	12128	1	1	39.9	38.3
12	940	946	11188	1	2	40.2	
11	93	1039	11095	3	5	38.9	
10	130	1169	10965	6	11	40.6	
9	556	1725	10409	10	21	40.8	
8	142	1867	10267	8	29	39.8	
7	919	2786	9348	7	36	37.8	
6	420	3206	8928	8	44	38.6	
5	475	3681	8453	4	48	39.0	
4	1719	5400	6734	7	55	36.5	
3	357	5757	6377	3	58	37.3	
1	25	5782	6352	1	59	40.2	

Table 14: HE

2.15 HU

Hierachy Level	Genes removed	Cum. sum of genes removed	Genes left	GO-terms removed	Cum. sum of GO-terms removed	Acc. (%)	Sig.acc. (%)
14	22	22	12106	2	2	47.5	46.8
13	319	341	11787	3	5	42.7	
12	857	1198	10930	4	9	46.3	
11	282	1480	10648	6	15	46.9	
10	758	2238	9890	6	21	47.5	
9	120	2358	9770	3	24	46.4	
8	461	2819	9309	15	39	48.2	
7	845	3664	8464	14	53	47.0	
6	179	3843	8285	10	63	47.3	
5	818	4661	7467	9	72	49.8	
4	150	4811	7317	5	77	49.7	
3	557	5368	6760	4	81	45.6	
2	344	5712	6416	1	82	46.0	

Table 15: HU

2.16 HUA

Hierachy Level	Genes removed	Cum. sum of genes removed	Genes left	GO-terms removed	Cum. sum of GO-terms removed	Acc. (%)	Sig.acc. (%)
17	36	36	10993	7	7	100.0	100.0
16	67	103	10926	11	18	100.0	
15	150	253	10776	22	40	99.9	
14	415	668	10361	74	114	100.0	
13	957	1625	9404	121	235	100.0	
12	1814	3439	7590	209	444	100.0	
11	1286	4725	6304	242	686	99.9	
10	1403	6128	4901	307	993	99.9	
9	1504	7632	3397	332	1325	100.0	

Table 16: HUA

2.17 IVSHINA

Hierachy Level	Genes removed	Cum. sum of genes removed	Genes left	GO-terms removed	Cum. sum of GO-terms removed	Acc. (%)	Sig.acc. (%)
14	21	21	12105	1	1	51.7	48.4
13	79	100	12026	2	3	48.5	
12	1342	1442	10684	7	10	49.1	
11	689	2131	9995	7	17	45.9	
10	380	2511	9615	7	24	48.0	
9	568	3079	9047	16	40	47.3	
8	286	3365	8761	25	65	47.0	
7	752	4117	8009	20	85	46.6	
6	353	4470	7656	15	100	45.3	
5	688	5158	6968	12	112	46.3	
4	436	5594	6532	13	125	46.6	
3	514	6108	6018	11	136	42.9	
2	109	6217	5909	6	142	46.8	
1	37	6254	5872	1	143	46.3	

Table 17: IVSHINA

2.18 KOK

Hierachy Level	Genes removed	Cum. sum of genes removed	Genes left	GO-terms removed	Cum. sum of GO-terms removed	Acc. (%)	Sig.acc. (%)
16	31	31	11962	2	2	93.4	94.9
15	89	120	11873	8	10	93.8	
14	165	285	11708	12	22	94.9	
13	689	974	11019	25	47	94.3	
12	1709	2683	9310	52	99	94.1	
11	1170	3853	8140	66	165	93.3	
10	1007	4860	7133	78	243	93.8	
9	1399	6259	5734	107	350	95.7	
8	1166	7425	4568	124	474	94.6	
7	1285	8710	3283	115	589	93.6	
6	714	9424	2569	107	696	95.0	
5	810	10234	1759	78	774	93.2	
4	473	10707	1286	51	825	97.3	
3	204	10911	1082	33	858	96.4	
2	202	11113	880	13	871	91.6	
1	14	11127	866	3	874	92.3	

Table 18: KOK

2.19 KORKOLA

Hierachy Level	Genes removed	Cum. sum of genes removed	Genes left	GO-terms removed	Cum. sum of GO-terms removed	Acc. (%)	Sig.acc. (%)
16	24	24	12096	2	2	55.5	54.2
15	5	29	12091	1	3	54.8	
14	55	84	12036	4	7	54.3	
13	363	447	11673	9	16	55.8	
12	1382	1829	10291	15	31	56.5	
11	665	2494	9626	17	48	55.2	
10	906	3400	8720	34	82	55.6	
9	905	4305	7815	33	115	57.7	
8	436	4741	7379	33	148	55.6	
7	1055	5796	6324	27	175	55.9	
6	784	6580	5540	39	214	56.0	
5	1037	7617	4503	29	243	55.5	
4	884	8501	3619	24	267	57.3	
3	317	8818	3302	13	280	58.2	
2	473	9291	2829	9	289	51.4	
1	69	9360	2760	2	291	52.5	

Table 19: KORKOLA

2.20 LIU

Hierachy Level	Genes removed	Cum. sum of genes removed	Genes left	GO-terms removed	Cum. sum of GO-terms removed	Acc. (%)	Sig.acc. (%)
15	98	98	11910	4	4	94.2	94.1
14	123	221	11787	12	16	93.6	
13	565	786	11222	29	45	92.3	
12	1852	2638	9370	54	99	93.2	
11	1209	3847	8161	81	180	92.4	
10	1277	5124	6884	90	270	92.5	
9	1238	6362	5646	109	379	94.5	
8	867	7229	4779	96	475	93.0	
7	1417	8646	3362	112	587	90.7	
6	666	9312	2696	111	698	89.2	
5	767	10079	1929	86	784	91.9	
4	549	10628	1380	44	828	95.7	
3	215	10843	1165	30	858	95.5	
2	238	11081	927	14	872	90.5	
1	9	11090	918	4	876	89.7	

Table 20: LIU

2.21 MA

Hierachy Level	Genes removed	Cum. sum of genes removed	Genes left	GO-terms removed	Cum. sum of GO-terms removed	Acc. (%)	Sig.acc. (%)
15	17	17	12094	1	1	62.0	61.9
14	41	58	12053	4	5	59.9	
13	293	351	11760	6	11	59.4	
12	1430	1781	10330	16	27	61.5	
11	691	2472	9639	25	52	61.4	
10	399	2871	9240	23	75	62.1	
9	1086	3957	8154	32	107	60.8	
8	404	4361	7750	29	136	63.5	
7	1015	5376	6735	41	177	59.8	
6	738	6114	5997	29	206	60.3	
5	790	6904	5207	18	224	56.9	
4	1169	8073	4038	13	237	58.0	
3	311	8384	3727	8	245	56.9	
2	445	8829	3282	6	251	54.3	
1	83	8912	3199	3	254	56.5	

Table 21: MA

2.22 MILLER

Hierachy Level	Genes removed	Cum. sum of genes removed	Genes left	GO-terms removed	Cum. sum of GO-terms removed	Acc. (%)	Sig.acc. (%)
14	33	33	12091	1	1	52.2	54.4
12	1390	1423	10701	6	7	52.6	
11	651	2074	10050	13	20	54.1	
10	337	2411	9713	12	32	50.7	
9	203	2614	9510	14	46	49.5	
8	328	2942	9182	16	62	52.3	
7	645	3587	8537	16	78	51.8	
6	561	4148	7976	22	100	51.2	
5	896	5044	7080	14	114	52.5	
4	1202	6246	5878	11	125	51.0	
3	829	7075	5049	11	136	51.1	
2	451	7526	4598	4	140	46.4	
1	165	7691	4433	2	142	48.4	

Table 22: MILLER

2.23 MORI

Hierachy Level	Genes removed	Cum. sum of genes removed	Genes left	GO-terms removed	Cum. sum of GO-terms removed	Acc. (%)	Sig.acc. (%)
16	24	24	12003	2	2	93.0	92.7
15	75	99	11928	8	10	93.7	
14	156	255	11772	12	22	92.3	
13	581	836	11191	17	39	92.0	
12	1880	2716	9311	41	80	93.5	
11	1063	3779	8248	51	131	93.3	
10	1077	4856	7171	76	207	92.9	
9	1222	6078	5949	87	294	94.3	
8	965	7043	4984	90	384	94.7	
7	1346	8389	3638	80	464	92.2	
6	725	9114	2913	94	558	92.8	
5	796	9910	2117	56	614	91.3	
4	564	10474	1553	39	653	94.7	
3	254	10728	1299	25	678	93.1	
2	278	11006	1021	15	693	85.7	
1	15	11021	1006	4	697	85.7	

Table 23: MORI

2.24 PAIK

Hierachy Level	Genes removed	Cum. sum of genes removed	Genes left	GO-terms removed	Cum. sum of GO-terms removed	Acc. (%)	Sig.acc. (%)
15	6	6	12118	2	2	49.7	52.3
14	75	81	12043	5	7	50.1	
13	239	320	11804	4	11	49.7	
12	1506	1826	10298	19	30	52.2	
11	846	2672	9452	22	52	47.1	
10	470	3142	8982	15	67	50.8	
9	1030	4172	7952	36	103	52.1	
8	349	4521	7603	26	129	51.4	
7	836	5357	6767	44	173	52.1	
6	811	6168	5956	53	226	48.7	
5	870	7038	5086	36	262	49.8	
4	1025	8063	4061	22	284	48.9	
3	341	8404	3720	20	304	49.6	
2	96	8500	3624	9	313	46.2	
1	17	8517	3607	2	315	44.5	

Table 24: PAIK

2.25 PAWITAN

Hierachy Level	Genes removed	Cum. sum of genes removed	Genes left	GO-terms removed	Cum. sum of GO-terms removed	Acc. (%)	Sig.acc. (%)
17	1	1	12100	1	1	67.1	65.0
15	12	13	12088	2	3	68.7	
14	144	157	11944	11	14	68.3	
13	317	474	11627	8	22	67.2	
12	1545	2019	10082	21	43	69.0	
11	787	2806	9295	33	76	68.4	
10	777	3583	8518	47	123	65.2	
9	643	4226	7875	45	168	66.6	
8	940	5166	6935	64	232	65.8	
7	1613	6779	5322	68	300	63.6	
6	453	7232	4869	54	354	64.7	
5	1088	8320	3781	48	402	64.1	
4	638	8958	3143	26	428	63.3	
3	217	9175	2926	16	444	63.7	
2	469	9644	2457	10	454	58.9	
1	12	9656	2445	2	456	57.5	

Table 25: PAWITAN

2.26 PEI

Hierachy Level	Genes removed	Cum. sum of genes removed	Genes left	GO-terms removed	Cum. sum of GO-terms removed	Acc. (%)	Sig.acc. (%)
16	3	3	12135	1	1	31.9	29.5
15	8	11	12127	3	4	30.4	
14	2	13	12125	1	5	28.5	
13	177	190	11948	2	7	28.0	
12	1318	1508	10630	9	16	30.5	
11	616	2124	10014	8	24	32.6	
10	432	2556	9582	10	34	27.7	
9	229	2785	9353	11	45	30.2	
8	190	2975	9163	13	58	27.8	
7	197	3172	8966	9	67	28.2	
6	533	3705	8433	24	91	30.0	
5	854	4559	7579	16	107	29.7	
4	837	5396	6742	4	111	30.7	
3	130	5526	6612	4	115	28.9	
2	250	5776	6362	3	118	27.8	
1	56	5832	6306	1	119	29.8	

Table 26: PEI

2.27 RAMASWAMY

Hierachy Level	Genes removed	Cum. sum of genes removed	Genes left	GO-terms removed	Cum. sum of GO-terms removed	Acc. (%)	Sig.acc. (%)
15	17	17	12109	1	1	51.4	48.8
14	8	25	12101	3	4	47.3	
13	58	83	12043	1	5	49.9	
12	1654	1737	10389	13	18	47.2	
11	768	2505	9621	9	27	48.6	
10	589	3094	9032	19	46	50.6	
9	263	3357	8769	8	54	48.6	
8	478	3835	8291	27	81	49.4	
7	1797	5632	6494	33	114	47.8	
6	548	6180	5946	38	152	49.7	
5	524	6704	5422	23	175	50.3	
4	905	7609	4517	18	193	48.1	
3	87	7696	4430	4	197	46.2	
2	209	7905	4221	6	203	47.3	
1	84	7989	4137	1	204	44.9	

Table 27: RAMASWAMY

2.28 REUTER

Hierachy Level	Genes removed	Cum. sum of genes removed	Genes left	GO-terms removed	Cum. sum of GO-terms removed	Acc. (%)	Sig.acc. (%)
17	14	14	11516	5	5	100.0	99.9
16	80	94	11436	6	11	99.9	
15	149	243	11287	22	33	99.8	
14	296	539	10991	42	75	99.9	
13	891	1430	10100	68	143	99.9	
12	1895	3325	8205	136	279	100.0	
11	1254	4579	6951	167	446	99.9	
10	1325	5904	5626	217	663	100.0	
9	1527	7431	4099	254	917	100.0	
8	1179	8610	2920	275	1192	100.0	
7	1033	9643	1887	220	1412	100.0	

Table 28: REUTER

2.29 RHODES

Hierachy Level	Genes removed	Cum. sum of genes removed	Genes left	GO-terms removed	Cum. sum of GO-terms removed	Acc. (%)	Sig.acc. (%)
16	31	31	12048	2	2	75.3	75.4
15	5	36	12043	1	3	75.8	
14	19	55	12024	2	5	78.0	
13	258	313	11766	9	14	75.6	
12	1642	1955	10124	24	38	74.9	
11	1141	3096	8983	47	85	76.0	
10	798	3894	8185	49	134	74.9	
9	1271	5165	6914	68	202	75.9	
8	1025	6190	5889	74	276	74.7	
7	1242	7432	4647	76	352	72.0	
6	736	8168	3911	68	420	73.9	
5	959	9127	2952	50	470	71.3	
4	655	9782	2297	27	497	73.8	
3	379	10161	1918	20	517	74.0	
2	394	10555	1524	12	529	74.1	
1	25	10580	1499	4	533	69.1	

Table 29: RHODES

2.30 SAAL

Hierachy Level	Genes removed	Cum. sum of genes removed	Genes left	GO-terms removed	Cum. sum of GO-terms removed	Acc. (%)	Sig.acc. (%)
18	17	17	11981	1	1	93.2	93.7
17	8	25	11973	2	3	93.4	
16	32	57	11941	4	7	92.9	
15	24	81	11917	6	13	93.9	
14	265	346	11652	19	32	92.5	
13	737	1083	10915	33	65	93.7	
12	1919	3002	8996	66	131	93.9	
11	1043	4045	7953	66	197	93.8	
10	1077	5122	6876	78	275	92.6	
9	1124	6246	5752	111	386	93.9	
8	1013	7259	4739	130	516	92.5	
7	1224	8483	3515	123	639	90.9	
6	693	9176	2822	110	749	90.8	
5	784	9960	2038	77	826	89.3	
4	536	10496	1502	47	873	94.6	
3	253	10749	1249	28	901	94.4	
2	269	11018	980	13	914	88.6	
1	24	11042	956	3	917	88.2	

Table 30: SAAL

2.31 SHIPITSIN

Hierachy Level	Genes removed	Cum. sum of genes removed	Genes left	GO-terms removed	Cum. sum of GO-terms removed	Acc. (%)	Sig.acc. (%)
16	24	24	12063	2	2	75.6	76.6
15	41	65	12022	4	6	74.6	
14	94	159	11928	8	14	75.7	
13	437	596	11491	15	29	73.1	
12	1701	2297	9790	33	62	72.9	
11	940	3237	8850	42	104	74.4	
10	889	4126	7961	45	149	75.1	
9	1095	5221	6866	64	213	77.2	
8	1090	6311	5776	75	288	73.2	
7	1437	7748	4339	71	359	74.6	
6	644	8392	3695	74	433	75.7	
5	830	9222	2865	47	480	73.0	
4	609	9831	2256	38	518	74.7	
3	281	10112	1975	13	531	73.1	
2	400	10512	1575	10	541	69.0	
1	23	10535	1552	5	546	66.9	

Table 31: SHIPITSIN

2.32 SORLIE

Hierachy Level	Genes removed	Cum. sum of genes removed	Genes left	GO-terms removed	Cum. sum of GO-terms removed	Acc. (%)	Sig.acc. (%)
16	25	25	12102	1	1	49.3	50.8
13	182	207	11920	2	3	47.5	
12	1523	1730	10397	7	10	51.1	
11	237	1967	10160	7	17	50.6	
10	772	2739	9388	13	30	51.2	
9	552	3291	8836	12	42	47.6	
8	345	3636	8491	19	61	49.8	
7	894	4530	7597	25	86	47.1	
6	591	5121	7006	22	108	53.6	
5	1136	6257	5870	22	130	50.3	
4	613	6870	5257	14	144	49.6	
3	393	7263	4864	9	153	52.1	
2	115	7378	4749	3	156	52.3	
1	24	7402	4725	2	158	53.4	

Table 32: SORLIE

2.33 SOTIRIOU-93

Hierachy Level	Genes removed	Cum. sum of genes removed	Genes left	GO-terms removed	Cum. sum of GO-terms removed	Acc. (%)	Sig.acc. (%)
16	74	74	11752	9	9	99.3	98.8
15	113	187	11639	12	21	99.5	
14	270	457	11369	28	49	99.3	
13	852	1309	10517	67	116	99.4	
12	1850	3159	8667	112	228	99.7	
11	1204	4363	7463	120	348	99.1	
10	1409	5772	6054	176	524	98.9	
9	1509	7281	4545	222	746	99.0	
8	1294	8575	3251	259	1005	98.3	
7	1020	9595	2231	198	1203	98.8	
6	631	10226	1600	177	1380	98.9	
5	516	10742	1084	95	1475	98.8	
4	307	11049	777	60	1535	99.8	

Table 33: SOTIRIOU-93

2.34 SOTIRIOU-GGI

Hierachy Level	Genes removed	Cum. sum of genes removed	Genes left	GO-terms removed	Cum. sum of GO-terms removed	Acc. (%)	Sig.acc. (%)
16	5	5	12046	1	1	86.0	85.1
15	5	10	12041	1	2	83.4	
14	32	42	12009	4	6	84.3	
13	437	479	11572	6	12	83.6	
12	1624	2103	9948	28	40	83.2	
11	1062	3165	8886	37	77	84.6	
10	677	3842	8209	45	122	82.2	
9	821	4663	7388	52	174	86.9	
8	772	5435	6616	69	243	82.1	
7	1711	7146	4905	83	326	81.8	
6	768	7914	4137	65	391	85.6	
5	852	8766	3285	37	428	85.6	
4	798	9564	2487	28	456	86.5	
3	196	9760	2291	24	480	83.8	
2	507	10267	1784	15	495	79.6	
1	24	10291	1760	2	497	80.0	

Table 34: SOTIRIOU-GGI

2.35 META-PCNA

Hierachy Level	Genes removed	Cum. sum of genes removed	Genes left	GO-terms removed	Cum. sum of GO-terms removed	Acc. (%)	Sig.acc. (%)
16	6	6	12014	1	1	89.1	90.4
15	31	37	11983	3	4	90.2	
14	21	58	11962	2	6	87.5	
13	522	580	11440	11	17	88.7	
12	1588	2168	9852	36	53	90.6	
11	1041	3209	8811	53	106	90.4	
10	975	4184	7836	58	164	89.8	
9	1263	5447	6573	76	240	92.1	
8	585	6032	5988	66	306	89.3	
7	1406	7438	4582	95	401	89.2	
6	760	8198	3822	82	483	90.1	
5	832	9030	2990	57	540	89.5	
4	809	9839	2181	35	575	92.3	
3	414	10253	1767	25	600	90.7	
2	346	10599	1421	17	617	87.9	
1	7	10606	1414	3	620	86.4	

Table 35: META-PCNA

2.36 TAUBE

Hierachy Level	Genes removed	Cum. sum of genes removed	Genes left	GO-terms removed	Cum. sum of GO-terms removed	Acc. (%)	Sig.acc. (%)
17	6	6	11930	2	2	98.0	98.1
16	66	72	11864	7	9	97.9	
15	80	152	11784	9	18	98.0	
14	171	323	11613	24	42	98.4	
13	641	964	10972	38	80	97.7	
12	1745	2709	9227	68	148	97.2	
11	924	3633	8303	77	225	98.2	
10	1105	4738	7198	109	334	98.4	
9	1442	6180	5756	150	484	98.1	
8	1154	7334	4602	156	640	97.7	
7	1243	8577	3359	135	775	97.1	
6	699	9276	2660	149	924	97.1	
5	663	9939	1997	94	1018	96.2	
4	460	10399	1537	54	1072	94.7	
3	183	10582	1354	26	1098	93.9	
2	215	10797	1139	11	1109	74.3	
1	21	10818	1118	4	1113	75.6	

Table 36: TAUBE

2.37 TAVAZOIE

Hierachy Level	Genes removed	Cum. sum of genes removed	Genes left	GO-terms removed	Cum. sum of GO-terms removed	Acc. (%)	Sig.acc. (%)
14	47	47	12088	1	1	37.9	37.3
13	126	173	11962	3	4	35.6	
12	906	1079	11056	2	6	39.6	
11	555	1634	10501	6	12	38.8	
10	660	2294	9841	14	26	37.3	
9	572	2866	9269	13	39	34.0	
8	228	3094	9041	16	55	38.0	
7	1573	4667	7468	24	79	36.9	
6	721	5388	6747	32	111	34.6	
5	1077	6465	5670	26	137	35.5	
4	554	7019	5116	15	152	39.4	
3	353	7372	4763	7	159	35.7	
2	130	7502	4633	2	161	37.1	

Table 37: TAVAZOIE

2.38 VALASTYAN

Hierachy Level	Genes removed	Cum. sum of genes removed	Genes left	GO-terms removed	Cum. sum of GO-terms removed	Acc. (%)	Sig.acc. (%)
14	69	69	12066	7	7	38.7	38.6
13	39	108	12027	4	11	39.7	
12	172	280	11855	6	17	40.2	
11	899	1179	10956	9	26	38.0	
10	318	1497	10638	13	39	40.7	
9	766	2263	9872	19	58	39.7	
8	667	2930	9205	33	91	38.5	
7	472	3402	8733	16	107	41.3	
6	422	3824	8311	24	131	37.2	
5	1118	4942	7193	22	153	37.8	
4	689	5631	6504	16	169	39.2	
3	404	6035	6100	7	176	37.2	
2	378	6413	5722	5	181	37.6	

Table 38: VALASTYAN

2.39 VANTVEER

Hierachy Level	Genes removed	Cum. sum of genes removed	Genes left	GO-terms removed	Cum. sum of GO-terms removed	Acc. (%)	Sig.acc. (%)
16	6	6	12082	1	1	73.3	74.0
15	24	30	12058	1	2	76.1	
14	111	141	11947	4	6	73.9	
13	525	666	11422	12	18	75.8	
12	1559	2225	9863	16	34	72.6	
11	880	3105	8983	31	65	75.9	
10	669	3774	8314	48	113	72.2	
9	1076	4850	7238	62	175	73.3	
8	639	5489	6599	49	224	71.1	
7	1545	7034	5054	60	284	69.4	
6	589	7623	4465	65	349	68.7	
5	881	8504	3584	51	400	70.9	
4	892	9396	2692	38	438	67.9	
3	384	9780	2308	15	453	66.6	
2	323	10103	1985	12	465	62.0	
1	29	10132	1956	2	467	61.9	

Table 39: VANTVEER

2.40 WANG-76

Hierachy Level	Genes removed	Cum. sum of genes removed	Genes left	GO-terms removed	Cum. sum of GO-terms removed	Acc. (%)	Sig.acc. (%)
17	6	6	12076	1	1	78.3	76.1
15	25	31	12051	3	4	77.5	
14	104	135	11947	7	11	76.9	
13	365	500	11582	12	23	74.4	
12	1649	2149	9933	27	50	76.7	
11	1048	3197	8885	38	88	77.7	
10	974	4171	7911	44	132	75.2	
9	1008	5179	6903	44	176	75.8	
8	1030	6209	5873	65	241	75.8	
7	1649	7858	4224	84	325	70.8	
6	643	8501	3581	62	387	71.2	
5	1001	9502	2580	46	433	74.4	
4	637	10139	1943	33	466	76.5	
3	295	10434	1648	19	485	73.8	
2	361	10795	1287	9	494	70.8	
1	17	10812	1270	3	497	68.2	

Table 40: WANG-76

2.41 WANG-ALK5T204D

Hierachy Level	Genes removed	Cum. sum of genes removed	Genes left	GO-terms removed	Cum. sum of GO-terms removed	Acc. (%)	Sig.acc. (%)
17	18	18	11946	2	2	96.8	97.3
16	43	61	11903	3	5	97.0	
15	76	137	11827	8	13	96.7	
14	239	376	11588	22	35	97.0	
13	687	1063	10901	35	70	96.7	
12	1790	2853	9111	57	127	96.8	
11	1254	4107	7857	83	210	97.6	
10	1151	5258	6706	98	308	96.9	
9	1465	6723	5241	146	454	96.6	
8	1189	7912	4052	159	613	96.4	
7	1219	9131	2833	148	761	94.5	
6	692	9823	2141	131	892	93.2	
5	609	10432	1532	86	978	92.1	
4	396	10828	1136	52	1030	96.3	
3	172	11000	964	26	1056	96.9	
2	233	11233	731	14	1070	91.8	
1	12	11245	719	3	1073	91.1	

Table 41: WANG-ALK5T204D

2.42 WELM

Hierachy Level	Genes removed	Cum. sum of genes removed	Genes left	GO-terms removed	Cum. sum of GO-terms removed	Acc. (%)	Sig.acc. (%)
12	54	54	12083	2	2	31.0	33.3
11	77	131	12006	2	4	32.3	
10	2	133	12004	1	5	33.2	
9	182	315	11822	3	8	32.0	
8	120	435	11702	4	12	34.9	
7	765	1200	10937	6	18	33.0	
6	370	1570	10567	3	21	36.1	
5	865	2435	9702	6	27	33.3	
4	1120	3555	8582	2	29	32.3	
3	32	3587	8550	1	30	34.4	
1	80	3667	8470	1	31	31.0	

Table 42: WELM

2.43 WEST

Hierachy Level	Genes removed	Cum. sum of genes removed	Genes left	GO-terms removed	Cum. sum of GO-terms removed	Acc. (%)	Sig.acc. (%)
16	68	68	11689	6	6	99.6	99.6
15	53	121	11636	9	15	99.9	
14	321	442	11315	34	49	99.7	
13	769	1211	10546	48	97	99.8	
12	1779	2990	8767	103	200	99.6	
11	1128	4118	7639	117	317	99.7	
10	1309	5427	6330	182	499	99.9	
9	1539	6966	4791	224	723	99.9	
8	1146	8112	3645	206	929	99.7	
7	1192	9304	2453	198	1127	99.8	
6	617	9921	1836	164	1291	99.7	
5	543	10464	1293	104	1395	99.7	
4	342	10806	951	66	1461	99.9	

Table 43: WEST

2.44 WHITFIELD

Hierachy Level	Genes removed	Cum. sum of genes removed	Genes left	GO-terms removed	Cum. sum of GO-terms removed	Acc. (%)	Sig.acc. (%)
16	18	18	11617	2	2	99.6	99.6
15	118	136	11499	15	17	99.9	
14	261	397	11238	31	48	99.4	
13	965	1362	10273	78	126	99.8	
12	1908	3270	8365	123	249	99.7	
11	1172	4442	7193	155	404	99.8	
10	1337	5779	5856	207	611	99.8	
9	1558	7337	4298	225	836	100.0	
8	1155	8492	3143	244	1080	99.9	
7	1005	9497	2138	211	1291	99.8	
6	621	10118	1517	181	1472	99.9	

Table 44: WHITFIELD

2.45 WONG-ESC

Hierachy Level	Genes removed	Cum. sum of genes removed	Genes left	GO-terms removed	Cum. sum of GO-terms removed	Acc. (%)	Sig.acc. (%)
16	30	30	11807	2	2	99.0	99.2
15	50	80	11757	7	9	99.0	
14	219	299	11538	20	29	99.2	
13	772	1071	10766	47	76	99.0	
12	1733	2804	9033	77	153	99.5	
11	1218	4022	7815	114	267	99.1	
10	1337	5359	6478	148	415	99.0	
9	1308	6667	5170	164	579	98.7	
8	941	7608	4229	145	724	97.9	
7	1171	8779	3058	157	881	97.9	
6	633	9412	2425	116	997	96.9	
5	812	10224	1613	82	1079	94.6	
4	458	10682	1155	50	1129	99.7	
3	216	10898	939	26	1155	98.9	
2	185	11083	754	14	1169	97.2	
1	14	11097	740	4	1173	95.9	

Table 45: WONG-ESC

2.46 WONG-MITOCHON

Hierachy Level	Genes removed	Cum. sum of genes removed	Genes left	GO-terms removed	Cum. sum of GO-terms removed	Acc. (%)	Sig.acc. (%)
18	17	17	11913	1	1	98.5	97.5
17	9	26	11904	3	4	98.1	
14	63	89	11841	9	13	97.7	
13	228	317	11613	12	25	98.3	
12	1228	1545	10385	25	50	98.2	
11	826	2371	9559	53	103	97.9	
10	1054	3425	8505	60	163	97.4	
9	1032	4457	7473	70	233	96.5	
8	949	5406	6524	77	310	97.6	
7	1754	7160	4770	89	399	96.9	
6	810	7970	3960	78	477	96.2	
5	868	8838	3092	67	544	94.9	
4	461	9299	2631	35	579	95.8	
3	339	9638	2292	24	603	96.6	
2	238	9876	2054	12	615	94.1	
1	30	9906	2024	3	618	95.2	

Table 46: WONG-MITOCHON

2.47 WONG-PROTEAS

Hierachy Level	Genes removed	Cum. sum of genes removed	Genes left	GO-terms removed	Cum. sum of GO-terms removed	Acc. (%)	Sig.acc. (%)
15	6	6	12090	1	1	73.1	73.3
14	33	39	12057	3	4	71.1	
13	317	356	11740	7	11	71.3	
12	1368	1724	10372	8	19	72.5	
11	874	2598	9498	18	37	71.3	
10	769	3367	8729	26	63	68.7	
9	838	4205	7891	22	85	71.1	
8	249	4454	7642	20	105	70.2	
7	1112	5566	6530	22	127	68.4	
6	264	5830	6266	20	147	67.4	
5	750	6580	5516	20	167	70.1	
4	785	7365	4731	13	180	69.6	
3	250	7615	4481	11	191	68.3	
2	140	7755	4341	3	194	66.0	
1	73	7828	4268	4	198	65.3	

Table 47: WONG-PROTEAS

2.48 YU

Hierachy Level	Genes removed	Cum. sum of genes removed	Genes left	GO-terms removed	Cum. sum of GO-terms removed	Acc. (%)	Sig.acc. (%)
16	32	32	12094	3	3	44.7	47.2
14	8	40	12086	2	5	48.9	
13	382	422	11704	7	12	52.2	
12	1396	1818	10308	12	24	48.5	
11	341	2159	9967	15	39	53.8	
10	186	2345	9781	24	63	50.5	
9	1108	3453	8673	34	97	48.9	
8	329	3782	8344	27	124	50.8	
7	1252	5034	7092	37	161	53.3	
6	478	5512	6614	39	200	48.0	
5	929	6441	5685	35	235	51.7	
4	626	7067	5059	22	257	48.4	
3	397	7464	4662	14	271	52.5	
2	447	7911	4215	5	276	53.9	
1	48	7959	4167	1	277	51.3	

Table 48: YU

3 GRP 2 with Bonferroni correction

Results for GRP 2. The p-values are Bonferroni corrected. The Sig.acc.(%) column gives the accuracy obtained when only the initial genes in the signature have been omitted. The results in all other columns are for GRP 2.

3.1 ABBA

Hierachy Level	Genes removed	Cum. sum of genes removed	Genes left	GO-terms removed	Cum. sum of GO-terms removed	Acc. (%)	Sig.acc. (%)
16	8	8	12027	2	2	40.9	44.9
15	13	21	12014	5	7	41.8	
14	129	150	11885	12	19	43.1	
13	473	623	11412	19	38	41.7	
12	1808	2431	9604	45	83	42.2	
11	1104	3535	8500	65	148	45.3	
10	927	4462	7573	70	218	44.0	
9	1267	5729	6306	100	318	42.8	
8	948	6677	5358	102	420	40.9	
7	1301	7978	4057	108	528	34.7	
6	655	8633	3402	94	622	37.6	
5	869	9502	2533	67	689	33.7	
4	712	10214	1821	39	728	29.3	
3	260	10474	1561	23	751	25.8	
2	319	10793	1242	16	767	21.4	
1	16	10809	1226	4	771	22.7	

Table 49: ABBA

3.2 ADORNO

Hierachy Level	Genes removed	Cum. sum of genes removed	Genes left	GO-terms removed	Cum. sum of GO-terms removed	Acc. (%)	Sig.acc. (%)
12	697	697	11441	2	2	3.4	2.0
11	277	974	11164	2	4	3.2	
10	615	1589	10549	1	5	3.6	
8	18	1607	10531	2	7	2.7	
7	464	2071	10067	1	8	2.6	
6	101	2172	9966	3	11	3.5	
4	46	2218	9920	1	12	2.8	
3	375	2593	9545	1	13	4.1	
2	363	2956	9182	3	16	2.8	
1	107	3063	9075	2	18	2.9	

Table 50: ADORNO

3.3 BEN-PORATH-EXP1

Hierachy Level	Genes removed	Cum. sum of genes removed	Genes left	GO-terms removed	Cum. sum of GO-terms removed	Acc. (%)	Sig.acc. (%)
18	17	17	11796	1	1	89.3	89.8
17	1	18	11795	1	2	90.5	
16	39	57	11756	3	5	89.3	
15	91	148	11665	13	18	91.4	
14	301	449	11364	29	47	89.1	
13	861	1310	10503	55	102	89.3	
12	1854	3164	8649	95	197	88.1	
11	1125	4289	7524	121	318	85.3	
10	1286	5575	6238	177	495	82.1	
9	1574	7149	4664	197	692	90.2	
8	1093	8242	3571	188	880	85.0	
7	1138	9380	2433	170	1050	87.0	
6	658	10038	1775	151	1201	78.9	
5	605	10643	1170	101	1302	77.0	
4	348	10991	822	46	1348	90.3	

Table 51: BEN-PORATH-EXP1

3.4 BEN-PORATH-PRC2

Hierachy Level	Genes removed	Cum. sum of genes removed	Genes left	GO-terms removed	Cum. sum of GO-terms removed	Acc. (%)	Sig.acc. (%)
17	12	12	11678	4	4	96.1	95.9
16	59	71	11619	5	9	95.6	
15	128	199	11491	20	29	97.1	
14	284	483	11207	39	68	95.9	
13	833	1316	10374	69	137	96.6	
12	1657	2973	8717	110	247	96.9	
11	1002	3975	7715	136	383	94.6	
10	1151	5126	6564	158	541	96.4	
9	1472	6598	5092	222	763	97.3	
8	1136	7734	3956	208	971	98.6	
7	1124	8858	2832	181	1152	97.5	
6	719	9577	2113	179	1331	97.9	
5	577	10154	1536	113	1444	98.0	

Table 52: BEN-PORATH-PRC2

3.5 BUESS

Hierachy Level	Genes removed	Cum. sum of genes removed	Genes left	GO-terms removed	Cum. sum of GO-terms removed	Acc. (%)	Sig.acc. (%)
16	25	25	12093	1	1	16.4	14.4
14	3	28	12090	2	3	17.5	
13	296	324	11794	10	13	15.4	
12	1389	1713	10405	14	27	16.1	
11	606	2319	9799	17	44	16.3	
10	642	2961	9157	17	61	14.6	
9	595	3556	8562	27	88	13.0	
8	315	3871	8247	19	107	13.2	
7	1513	5384	6734	36	143	13.4	
6	590	5974	6144	29	172	14.0	
5	723	6697	5421	24	196	12.7	
4	627	7324	4794	16	212	12.5	
3	246	7570	4548	5	217	13.7	
2	47	7617	4501	3	220	13.2	
1	82	7699	4419	2	222	12.8	

Table 53: BUESS

3.6 BUFFA

Hierachy Level	Genes removed	Cum. sum of genes removed	Genes left	GO-terms removed	Cum. sum of GO-terms removed	Acc. (%)	Sig.acc. (%)
16	24	24	12114	3	3	3.4	4.3
15	26	50	12088	2	5	4.2	
14	29	79	12059	4	9	3.1	
13	143	222	11916	5	14	4.0	
12	1424	1646	10492	10	24	4.0	
11	497	2143	9995	9	33	3.6	
10	212	2355	9783	14	47	4.3	
9	462	2817	9321	10	57	4.7	
8	161	2978	9160	9	66	4.5	
7	544	3522	8616	17	83	2.9	
6	262	3784	8354	11	94	3.9	
5	422	4206	7932	10	104	3.1	
4	1088	5294	6844	7	111	3.6	
3	337	5631	6507	3	114	4.3	
2	1	5632	6506	1	115	4.5	

Table 54: BUFFA

3.7 CARTER

Hierachy Level	Genes removed	Cum. sum of genes removed	Genes left	GO-terms removed	Cum. sum of GO-terms removed	Acc. (%)	Sig.acc. (%)
16	6	6	12065	1	1	32.9	34.9
15	29	35	12036	2	3	34.6	
14	62	97	11974	3	6	32.3	
13	286	383	11688	9	15	34.0	
12	1554	1937	10134	30	45	32.1	
11	983	2920	9151	32	77	31.8	
10	538	3458	8613	34	111	29.0	
9	1368	4826	7245	65	176	27.4	
8	544	5370	6701	54	230	29.7	
7	1178	6548	5523	65	295	24.2	
6	595	7143	4928	51	346	25.4	
5	1068	8211	3860	36	382	27.1	
4	558	8769	3302	26	408	26.3	
3	466	9235	2836	23	431	23.2	
2	322	9557	2514	10	441	23.4	
1	109	9666	2405	4	445	23.0	

Table 55: CARTER

3.8 CHANG

Hierachy Level	Genes removed	Cum. sum of genes removed	Genes left	GO-terms removed	Cum. sum of GO-terms removed	Acc. (%)	Sig.acc. (%)
18	17	17	11822	1	1	87.0	88.5
17	11	28	11811	3	4	87.9	
16	3	31	11808	2	6	89.6	
15	89	120	11719	8	14	89.2	
14	203	323	11516	24	38	89.8	
13	819	1142	10697	41	79	87.4	
12	1932	3074	8765	91	170	87.1	
11	1246	4320	7519	112	282	84.8	
10	1216	5536	6303	153	435	84.3	
9	1326	6862	4977	174	609	81.9	
8	1252	8114	3725	183	792	79.6	
7	1258	9372	2467	193	985	70.8	
6	711	10083	1756	165	1150	81.2	
5	573	10656	1183	97	1247	73.2	
4	336	10992	847	54	1301	88.1	
3	122	11114	725	27	1328	86.3	

Table 56: CHANG

3.9 CHI

Hierachy Level	Genes removed	Cum. sum of genes removed	Genes left	GO-terms removed	Cum. sum of GO-terms removed	Acc. (%)	Sig.acc. (%)
16	20	20	12014	1	1	50.9	54.1
15	56	76	11958	4	5	51.4	
14	191	267	11767	13	18	52.6	
13	553	820	11214	18	36	53.1	
12	1642	2462	9572	35	71	54.0	
11	939	3401	8633	50	121	54.0	
10	1070	4471	7563	65	186	53.6	
9	1077	5548	6486	76	262	55.0	
8	1086	6634	5400	96	358	57.6	
7	1625	8259	3775	99	457	53.3	
6	648	8907	3127	92	549	52.6	
5	821	9728	2306	72	621	53.7	
4	536	10264	1770	49	670	55.8	
3	229	10493	1541	25	695	54.4	
2	255	10748	1286	14	709	41.5	
1	13	10761	1273	3	712	41.5	

Table 57: CHI

3.10 CRAWFORD

Hierachy Level	Genes removed	Cum. sum of genes removed	Genes left	GO-terms removed	Cum. sum of GO-terms removed	Acc. (%)	Sig.acc. (%)
16	26	26	11791	3	3	82.9	82.8
15	90	116	11701	11	14	84.1	
14	218	334	11483	24	38	83.5	
13	925	1259	10558	57	95	80.9	
12	1858	3117	8700	88	183	83.0	
11	1127	4244	7573	125	308	79.3	
10	1281	5525	6292	163	471	79.0	
9	1456	6981	4836	196	667	75.0	
8	1139	8120	3697	190	857	70.0	
7	1120	9240	2577	174	1031	74.7	
6	670	9910	1907	150	1181	75.9	
5	643	10553	1264	99	1280	64.7	
4	376	10929	888	56	1336	75.5	

Table 58: CRAWFORD

3.11 DAI

Hierachy Level	Genes removed	Cum. sum of genes removed	Genes left	GO-terms removed	Cum. sum of GO-terms removed	Acc. (%)	Sig.acc. (%)
16	6	6	12099	1	1	19.4	18.2
14	18	24	12081	1	2	17.3	
13	150	174	11931	5	7	18.5	
12	1388	1562	10543	9	16	16.9	
11	909	2471	9634	23	39	18.6	
10	602	3073	9032	19	58	16.9	
9	980	4053	8052	36	94	17.1	
8	435	4488	7617	25	119	14.8	
7	1137	5625	6480	42	161	16.3	
6	769	6394	5711	34	195	15.4	
5	728	7122	4983	20	215	14.5	
4	1238	8360	3745	15	230	14.3	
3	299	8659	3446	11	241	16.0	
2	432	9091	3014	7	248	12.6	
1	37	9128	2977	2	250	11.4	

Table 59: DAI

3.12 GLINSKY

Hierachy Level	Genes removed	Cum. sum of genes removed	Genes left	GO-terms removed	Cum. sum of GO-terms removed	Acc. (%)	Sig.acc. (%)
16	6	6	12124	1	1	6.9	9.3
15	6	12	12118	2	3	9.3	
14	27	39	12091	2	5	7.6	
13	255	294	11836	6	11	6.9	
12	1503	1797	10333	12	23	9.7	
11	384	2181	9949	17	40	8.9	
10	615	2796	9334	18	58	8.3	
9	658	3454	8676	18	76	9.1	
8	198	3652	8478	16	92	8.6	
7	684	4336	7794	26	118	7.1	
6	513	4849	7281	26	144	6.5	
5	839	5688	6442	21	165	5.9	
4	1162	6850	5280	17	182	7.1	
3	137	6987	5143	5	187	7.0	
2	110	7097	5033	3	190	6.3	
1	26	7123	5007	1	191	6.9	

Table 60: GLINSKY

3.13 HALLSTROM

Hierachy Level	Genes removed	Cum. sum of genes removed	Genes left	GO-terms removed	Cum. sum of GO-terms removed	Acc. (%)	Sig.acc. (%)
15	69	69	12009	4	4	33.6	32.1
14	64	133	11945	4	8	33.0	
13	463	596	11482	13	21	31.6	
12	1547	2143	9935	24	45	32.6	
11	826	2969	9109	32	77	30.6	
10	909	3878	8200	51	128	32.2	
9	1195	5073	7005	54	182	33.7	
8	841	5914	6164	66	248	35.4	
7	1711	7625	4453	76	324	31.8	
6	593	8218	3860	67	391	34.0	
5	1024	9242	2836	48	439	37.4	
4	582	9824	2254	26	465	36.6	
3	220	10044	2034	16	481	34.2	
2	323	10367	1711	9	490	32.5	
1	21	10388	1690	4	494	28.9	

Table 61: HALLSTROM

3.14 HE

Hierachy Level	Genes removed	Cum. sum of genes removed	Genes left	GO-terms removed	Cum. sum of GO-terms removed	Acc. (%)	Sig.acc. (%)
15	6	6	12128	1	1	6.9	5.9
12	940	946	11188	1	2	6.3	
11	93	1039	11095	3	5	5.7	
10	130	1169	10965	6	11	7.5	
9	556	1725	10409	10	21	7.2	
8	142	1867	10267	8	29	6.3	
7	919	2786	9348	7	36	5.6	
6	420	3206	8928	8	44	6.6	
5	475	3681	8453	4	48	5.9	
4	1719	5400	6734	7	55	6.7	
3	357	5757	6377	3	58	7.2	
1	25	5782	6352	1	59	7.1	

Table 62: HE

3.15 HU

Hierachy Level	Genes removed	Cum. sum of genes removed	Genes left	GO-terms removed	Cum. sum of GO-terms removed	Acc. (%)	Sig.acc. (%)
14	22	22	12106	2	2	10.7	9.1
13	319	341	11787	3	5	9.9	
12	857	1198	10930	4	9	10.5	
11	282	1480	10648	6	15	9.6	
10	758	2238	9890	6	21	9.1	
9	120	2358	9770	3	24	9.2	
8	461	2819	9309	15	39	11.1	
7	845	3664	8464	14	53	10.1	
6	179	3843	8285	10	63	9.3	
5	818	4661	7467	9	72	10.5	
4	150	4811	7317	5	77	11.4	
3	557	5368	6760	4	81	11.0	
2	344	5712	6416	1	82	8.8	

Table 63: HU

3.16 HUA

Hierachy Level	Genes removed	Cum. sum of genes removed	Genes left	GO-terms removed	Cum. sum of GO-terms removed	Acc. (%)	Sig.acc. (%)
17	36	36	10993	7	7	100.0	99.9
16	67	103	10926	11	18	99.8	
15	150	253	10776	22	40	99.4	
14	415	668	10361	74	114	99.6	
13	957	1625	9404	121	235	99.9	
12	1814	3439	7590	209	444	98.8	
11	1286	4725	6304	242	686	98.2	
10	1403	6128	4901	307	993	98.8	
9	1504	7632	3397	332	1325	99.7	

Table 64: HUA

3.17 IVSHINA

Hierachy Level	Genes removed	Cum. sum of genes removed	Genes left	GO-terms removed	Cum. sum of GO-terms removed	Acc. (%)	Sig.acc. (%)
14	21	21	12105	1	1	11.4	11.1
13	79	100	12026	2	3	9.0	
12	1342	1442	10684	7	10	11.0	
11	689	2131	9995	7	17	9.3	
10	380	2511	9615	7	24	7.2	
9	568	3079	9047	16	40	8.3	
8	286	3365	8761	25	65	9.1	
7	752	4117	8009	20	85	8.2	
6	353	4470	7656	15	100	8.5	
5	688	5158	6968	12	112	7.0	
4	436	5594	6532	13	125	7.7	
3	514	6108	6018	11	136	7.5	
2	109	6217	5909	6	142	6.1	
1	37	6254	5872	1	143	7.2	

Table 65: IVSHINA

3.18 KOK

Hierachy Level	Genes removed	Cum. sum of genes removed	Genes left	GO-terms removed	Cum. sum of GO-terms removed	Acc. (%)	Sig.acc. (%)
16	31	31	11962	2	2	61.9	62.2
15	89	120	11873	8	10	62.1	
14	165	285	11708	12	22	64.0	
13	689	974	11019	25	47	58.3	
12	1709	2683	9310	52	99	63.7	
11	1170	3853	8140	66	165	60.0	
10	1007	4860	7133	78	243	55.9	
9	1399	6259	5734	107	350	64.0	
8	1166	7425	4568	124	474	61.0	
7	1285	8710	3283	115	589	58.8	
6	714	9424	2569	107	696	62.2	
5	810	10234	1759	78	774	62.6	
4	473	10707	1286	51	825	62.6	
3	204	10911	1082	33	858	57.6	
2	202	11113	880	13	871	51.1	
1	14	11127	866	3	874	50.0	

Table 66: KOK

3.19 KORKOLA

Hierachy Level	Genes removed	Cum. sum of genes removed	Genes left	GO-terms removed	Cum. sum of GO-terms removed	Acc. (%)	Sig.acc. (%)
16	24	24	12096	2	2	13.5	14.0
15	5	29	12091	1	3	12.3	
14	55	84	12036	4	7	13.5	
13	363	447	11673	9	16	12.8	
12	1382	1829	10291	15	31	14.6	
11	665	2494	9626	17	48	13.3	
10	906	3400	8720	34	82	13.1	
9	905	4305	7815	33	115	13.6	
8	436	4741	7379	33	148	13.3	
7	1055	5796	6324	27	175	12.8	
6	784	6580	5540	39	214	12.9	
5	1037	7617	4503	29	243	12.4	
4	884	8501	3619	24	267	14.4	
3	317	8818	3302	13	280	14.6	
2	473	9291	2829	9	289	9.9	
1	69	9360	2760	2	291	10.6	

Table 67: KORKOLA

3.20 LIU

Hierachy Level	Genes removed	Cum. sum of genes removed	Genes left	GO-terms removed	Cum. sum of GO-terms removed	Acc. (%)	Sig.acc. (%)
15	98	98	11910	4	4	60.7	61.1
14	123	221	11787	12	16	59.9	
13	565	786	11222	29	45	56.6	
12	1852	2638	9370	54	99	56.5	
11	1209	3847	8161	81	180	56.7	
10	1277	5124	6884	90	270	57.8	
9	1238	6362	5646	109	379	58.7	
8	867	7229	4779	96	475	57.9	
7	1417	8646	3362	112	587	52.5	
6	666	9312	2696	111	698	45.4	
5	767	10079	1929	86	784	52.1	
4	549	10628	1380	44	828	53.9	
3	215	10843	1165	30	858	52.5	
2	238	11081	927	14	872	25.2	
1	9	11090	918	4	876	25.2	

Table 68: LIU

3.21 MA

Hierachy Level	Genes removed	Cum. sum of genes removed	Genes left	GO-terms removed	Cum. sum of GO-terms removed	Acc. (%)	Sig.acc. (%)
15	17	17	12094	1	1	15.2	17.5
14	41	58	12053	4	5	17.3	
13	293	351	11760	6	11	18.2	
12	1430	1781	10330	16	27	16.4	
11	691	2472	9639	25	52	16.1	
10	399	2871	9240	23	75	17.7	
9	1086	3957	8154	32	107	16.8	
8	404	4361	7750	29	136	15.2	
7	1015	5376	6735	41	177	13.6	
6	738	6114	5997	29	206	12.8	
5	790	6904	5207	18	224	12.4	
4	1169	8073	4038	13	237	11.6	
3	311	8384	3727	8	245	10.2	
2	445	8829	3282	6	251	11.5	
1	83	8912	3199	3	254	8.9	

Table 69: MA

3.22 MILLER

Hierachy Level	Genes removed	Cum. sum of genes removed	Genes left	GO-terms removed	Cum. sum of GO-terms removed	Acc. (%)	Sig.acc. (%)
14	33	33	12091	1	1	12.5	14.0
12	1390	1423	10701	6	7	11.4	
11	651	2074	10050	13	20	11.2	
10	337	2411	9713	12	32	12.7	
9	203	2614	9510	14	46	9.9	
8	328	2942	9182	16	62	10.6	
7	645	3587	8537	16	78	11.5	
6	561	4148	7976	22	100	11.5	
5	896	5044	7080	14	114	11.2	
4	1202	6246	5878	11	125	13.3	
3	829	7075	5049	11	136	11.2	
2	451	7526	4598	4	140	9.6	
1	165	7691	4433	2	142	9.0	

Table 70: MILLER

3.23 MORI

Hierachy Level	Genes removed	Cum. sum of genes removed	Genes left	GO-terms removed	Cum. sum of GO-terms removed	Acc. (%)	Sig.acc. (%)
16	24	24	12003	2	2	56.7	56.9
15	75	99	11928	8	10	58.6	
14	156	255	11772	12	22	58.4	
13	581	836	11191	17	39	60.0	
12	1880	2716	9311	41	80	56.1	
11	1063	3779	8248	51	131	57.8	
10	1077	4856	7171	76	207	55.6	
9	1222	6078	5949	87	294	59.3	
8	965	7043	4984	90	384	60.9	
7	1346	8389	3638	80	464	54.3	
6	725	9114	2913	94	558	52.3	
5	796	9910	2117	56	614	53.4	
4	564	10474	1553	39	653	53.1	
3	254	10728	1299	25	678	46.5	
2	278	11006	1021	15	693	30.9	
1	15	11021	1006	4	697	27.2	

Table 71: MORI

3.24 PAIK

Hierachy Level	Genes removed	Cum. sum of genes removed	Genes left	GO-terms removed	Cum. sum of GO-terms removed	Acc. (%)	Sig.acc. (%)
15	6	6	12118	2	2	10.0	10.4
14	75	81	12043	5	7	9.9	
13	239	320	11804	4	11	11.1	
12	1506	1826	10298	19	30	13.2	
11	846	2672	9452	22	52	10.8	
10	470	3142	8982	15	67	10.7	
9	1030	4172	7952	36	103	10.3	
8	349	4521	7603	26	129	10.5	
7	836	5357	6767	44	173	10.7	
6	811	6168	5956	53	226	9.6	
5	870	7038	5086	36	262	10.3	
4	1025	8063	4061	22	284	10.4	
3	341	8404	3720	20	304	11.6	
2	96	8500	3624	9	313	7.9	
1	17	8517	3607	2	315	7.8	

Table 72: PAIK

3.25 PAWITAN

Hierachy Level	Genes removed	Cum. sum of genes removed	Genes left	GO-terms removed	Cum. sum of GO-terms removed	Acc. (%)	Sig.acc. (%)
17	1	1	12100	1	1	21.9	21.1
15	12	13	12088	2	3	23.8	
14	144	157	11944	11	14	22.9	
13	317	474	11627	8	22	22.0	
12	1545	2019	10082	21	43	22.6	
11	787	2806	9295	33	76	21.5	
10	777	3583	8518	47	123	22.1	
9	643	4226	7875	45	168	21.6	
8	940	5166	6935	64	232	20.4	
7	1613	6779	5322	68	300	17.0	
6	453	7232	4869	54	354	17.4	
5	1088	8320	3781	48	402	18.2	
4	638	8958	3143	26	428	18.8	
3	217	9175	2926	16	444	17.3	
2	469	9644	2457	10	454	16.0	
1	12	9656	2445	2	456	14.0	

Table 73: PAWITAN

3.26 PEI

Hierachy Level	Genes removed	Cum. sum of genes removed	Genes left	GO-terms removed	Cum. sum of GO-terms removed	Acc. (%)	Sig.acc. (%)
16	3	3	12135	1	1	3.3	2.5
15	8	11	12127	3	4	4.0	
14	2	13	12125	1	5	2.9	
13	177	190	11948	2	7	4.7	
12	1318	1508	10630	9	16	3.3	
11	616	2124	10014	8	24	3.7	
10	432	2556	9582	10	34	3.2	
9	229	2785	9353	11	45	3.4	
8	190	2975	9163	13	58	3.1	
7	197	3172	8966	9	67	2.8	
6	533	3705	8433	24	91	2.5	
5	854	4559	7579	16	107	3.2	
4	837	5396	6742	4	111	4.1	
3	130	5526	6612	4	115	2.5	
2	250	5776	6362	3	118	3.0	
1	56	5832	6306	1	119	3.2	

Table 74: PEI

3.27 RAMASWAMY

Hierachy Level	Genes removed	Cum. sum of genes removed	Genes left	GO-terms removed	Cum. sum of GO-terms removed	Acc. (%)	Sig.acc. (%)
15	17	17	12109	1	1	9.7	11.5
14	8	25	12101	3	4	9.8	
13	58	83	12043	1	5	10.4	
12	1654	1737	10389	13	18	11.4	
11	768	2505	9621	9	27	8.3	
10	589	3094	9032	19	46	11.5	
9	263	3357	8769	8	54	11.7	
8	478	3835	8291	27	81	9.9	
7	1797	5632	6494	33	114	8.0	
6	548	6180	5946	38	152	9.2	
5	524	6704	5422	23	175	8.5	
4	905	7609	4517	18	193	9.1	
3	87	7696	4430	4	197	7.3	
2	209	7905	4221	6	203	7.1	
1	84	7989	4137	1	204	8.1	

Table 75: RAMASWAMY

3.28 REUTER

Hierachy Level	Genes removed	Cum. sum of genes removed	Genes left	GO-terms removed	Cum. sum of GO-terms removed	Acc. (%)	Sig.acc. (%)
17	14	14	11516	5	5	99.1	98.8
16	80	94	11436	6	11	98.6	
15	149	243	11287	22	33	99.2	
14	296	539	10991	42	75	98.7	
13	891	1430	10100	68	143	99.0	
12	1895	3325	8205	136	279	98.4	
11	1254	4579	6951	167	446	97.8	
10	1325	5904	5626	217	663	99.2	
9	1527	7431	4099	254	917	99.8	
8	1179	8610	2920	275	1192	99.5	
7	1033	9643	1887	220	1412	99.1	

Table 76: REUTER

3.29 RHODES

Hierachy Level	Genes removed	Cum. sum of genes removed	Genes left	GO-terms removed	Cum. sum of GO-terms removed	Acc. (%)	Sig.acc. (%)
16	31	31	12048	2	2	30.0	28.8
15	5	36	12043	1	3	32.4	
14	19	55	12024	2	5	32.6	
13	258	313	11766	9	14	30.5	
12	1642	1955	10124	24	38	30.0	
11	1141	3096	8983	47	85	29.8	
10	798	3894	8185	49	134	26.9	
9	1271	5165	6914	68	202	29.1	
8	1025	6190	5889	74	276	26.4	
7	1242	7432	4647	76	352	23.4	
6	736	8168	3911	68	420	23.1	
5	959	9127	2952	50	470	24.8	
4	655	9782	2297	27	497	23.0	
3	379	10161	1918	20	517	21.6	
2	394	10555	1524	12	529	21.6	
1	25	10580	1499	4	533	20.0	

Table 77: RHODES

3.30 SAAL

Hierachy Level	Genes removed	Cum. sum of genes removed	Genes left	GO-terms removed	Cum. sum of GO-terms removed	Acc. (%)	Sig.acc. (%)
18	17	17	11981	1	1	57.9	61.2
17	8	25	11973	2	3	59.0	
16	32	57	11941	4	7	59.9	
15	24	81	11917	6	13	59.7	
14	265	346	11652	19	32	60.3	
13	737	1083	10915	33	65	57.8	
12	1919	3002	8996	66	131	60.1	
11	1043	4045	7953	66	197	54.4	
10	1077	5122	6876	78	275	52.7	
9	1124	6246	5752	111	386	57.6	
8	1013	7259	4739	130	516	52.1	
7	1224	8483	3515	123	639	45.5	
6	693	9176	2822	110	749	43.3	
5	784	9960	2038	77	826	41.4	
4	536	10496	1502	47	873	41.3	
3	253	10749	1249	28	901	37.5	
2	269	11018	980	13	914	22.6	
1	24	11042	956	3	917	20.4	

Table 78: SAAL

3.31 SHIPITSIN

Hierachy Level	Genes removed	Cum. sum of genes removed	Genes left	GO-terms removed	Cum. sum of GO-terms removed	Acc. (%)	Sig.acc. (%)
16	24	24	12063	2	2	28.5	30.7
15	41	65	12022	4	6	27.3	
14	94	159	11928	8	14	29.8	
13	437	596	11491	15	29	28.1	
12	1701	2297	9790	33	62	27.0	
11	940	3237	8850	42	104	29.9	
10	889	4126	7961	45	149	35.3	
9	1095	5221	6866	64	213	33.3	
8	1090	6311	5776	75	288	29.3	
7	1437	7748	4339	71	359	29.0	
6	644	8392	3695	74	433	31.8	
5	830	9222	2865	47	480	34.4	
4	609	9831	2256	38	518	31.5	
3	281	10112	1975	13	531	28.5	
2	400	10512	1575	10	541	20.4	
1	23	10535	1552	5	546	17.6	

Table 79: SHIPITSIN

3.32 SORLIE

Hierachy Level	Genes removed	Cum. sum of genes removed	Genes left	GO-terms removed	Cum. sum of GO-terms removed	Acc. (%)	Sig.acc. (%)
16	25	25	12102	1	1	9.1	11.1
13	182	207	11920	2	3	11.2	
12	1523	1730	10397	7	10	13.0	
11	237	1967	10160	7	17	11.3	
10	772	2739	9388	13	30	12.1	
9	552	3291	8836	12	42	10.2	
8	345	3636	8491	19	61	11.4	
7	894	4530	7597	25	86	9.5	
6	591	5121	7006	22	108	13.2	
5	1136	6257	5870	22	130	12.4	
4	613	6870	5257	14	144	12.2	
3	393	7263	4864	9	153	14.8	
2	115	7378	4749	3	156	13.1	
1	24	7402	4725	2	158	15.9	

Table 80: SORLIE

3.33 SOTIRIOU-93

Hierachy Level	Genes removed	Cum. sum of genes removed	Genes left	GO-terms removed	Cum. sum of GO-terms removed	Acc. (%)	Sig.acc. (%)
16	74	74	11752	9	9	86.0	88.3
15	113	187	11639	12	21	88.4	
14	270	457	11369	28	49	85.6	
13	852	1309	10517	67	116	87.5	
12	1850	3159	8667	112	228	88.8	
11	1204	4363	7463	120	348	83.8	
10	1409	5772	6054	176	524	81.6	
9	1509	7281	4545	222	746	81.7	
8	1294	8575	3251	259	1005	78.9	
7	1020	9595	2231	198	1203	76.9	
6	631	10226	1600	177	1380	76.3	
5	516	10742	1084	95	1475	80.8	
4	307	11049	777	60	1535	84.2	

Table 81: SOTIRIOU-93

3.34 SOTIRIOU-GGI

Hierachy Level	Genes removed	Cum. sum of genes removed	Genes left	GO-terms removed	Cum. sum of GO-terms removed	Acc. (%)	Sig.acc. (%)
16	5	5	12046	1	1	43.0	36.9
15	5	10	12041	1	2	40.5	
14	32	42	12009	4	6	38.1	
13	437	479	11572	6	12	36.9	
12	1624	2103	9948	28	40	39.8	
11	1062	3165	8886	37	77	34.5	
10	677	3842	8209	45	122	35.4	
9	821	4663	7388	52	174	38.3	
8	772	5435	6616	69	243	33.1	
7	1711	7146	4905	83	326	30.3	
6	768	7914	4137	65	391	33.5	
5	852	8766	3285	37	428	37.2	
4	798	9564	2487	28	456	32.9	
3	196	9760	2291	24	480	29.4	
2	507	10267	1784	15	495	27.0	
1	24	10291	1760	2	497	27.2	

Table 82: SOTIRIOU-GGI

3.35 META-PCNA

Hierachy Level	Genes removed	Cum. sum of genes removed	Genes left	GO-terms removed	Cum. sum of GO-terms removed	Acc. (%)	Sig.acc. (%)
16	6	6	12014	1	1	50.3	52.7
15	31	37	11983	3	4	50.8	
14	21	58	11962	2	6	48.4	
13	522	580	11440	11	17	47.3	
12	1588	2168	9852	36	53	49.2	
11	1041	3209	8811	53	106	48.2	
10	975	4184	7836	58	164	46.1	
9	1263	5447	6573	76	240	45.2	
8	585	6032	5988	66	306	44.2	
7	1406	7438	4582	95	401	39.1	
6	760	8198	3822	82	483	42.0	
5	832	9030	2990	57	540	40.3	
4	809	9839	2181	35	575	44.1	
3	414	10253	1767	25	600	39.4	
2	346	10599	1421	17	617	35.8	
1	7	10606	1414	3	620	30.1	

Table 83: META-PCNA

3.36 TAUBE

Hierachy Level	Genes removed	Cum. sum of genes removed	Genes left	GO-terms removed	Cum. sum of GO-terms removed	Acc. (%)	Sig.acc. (%)
17	6	6	11930	2	2	77.9	77.3
16	66	72	11864	7	9	77.3	
15	80	152	11784	9	18	76.9	
14	171	323	11613	24	42	79.2	
13	641	964	10972	38	80	79.1	
12	1745	2709	9227	68	148	78.5	
11	924	3633	8303	77	225	75.0	
10	1105	4738	7198	109	334	77.9	
9	1442	6180	5756	150	484	79.1	
8	1154	7334	4602	156	640	79.3	
7	1243	8577	3359	135	775	76.1	
6	699	9276	2660	149	924	76.2	
5	663	9939	1997	94	1018	77.3	
4	460	10399	1537	54	1072	73.1	
3	183	10582	1354	26	1098	65.8	
2	215	10797	1139	11	1109	35.5	
1	21	10818	1118	4	1113	32.5	

Table 84: TAUBE

3.37 TAVAZOIE

Hierachy Level	Genes removed	Cum. sum of genes removed	Genes left	GO-terms removed	Cum. sum of GO-terms removed	Acc. (%)	Sig.acc. (%)
14	47	47	12088	1	1	7.7	7.7
13	126	173	11962	3	4	5.5	
12	906	1079	11056	2	6	4.3	
11	555	1634	10501	6	12	5.9	
10	660	2294	9841	14	26	6.0	
9	572	2866	9269	13	39	4.7	
8	228	3094	9041	16	55	5.8	
7	1573	4667	7468	24	79	5.3	
6	721	5388	6747	32	111	5.6	
5	1077	6465	5670	26	137	4.1	
4	554	7019	5116	15	152	6.6	
3	353	7372	4763	7	159	6.0	
2	130	7502	4633	2	161	6.5	

Table 85: TAVAZOIE

3.38 VALASTYAN

Hierachy Level	Genes removed	Cum. sum of genes removed	Genes left	GO-terms removed	Cum. sum of GO-terms removed	Acc. (%)	Sig.acc. (%)
14	69	69	12066	7	7	6.1	5.3
13	39	108	12027	4	11	6.9	
12	172	280	11855	6	17	7.1	
11	899	1179	10956	9	26	5.2	
10	318	1497	10638	13	39	6.8	
9	766	2263	9872	19	58	7.9	
8	667	2930	9205	33	91	6.7	
7	472	3402	8733	16	107	7.3	
6	422	3824	8311	24	131	6.4	
5	1118	4942	7193	22	153	7.3	
4	689	5631	6504	16	169	7.3	
3	404	6035	6100	7	176	5.9	
2	378	6413	5722	5	181	5.4	

Table 86: VALASTYAN

3.39 VANTVEER

Hierachy Level	Genes removed	Cum. sum of genes removed	Genes left	GO-terms removed	Cum. sum of GO-terms removed	Acc. (%)	Sig.acc. (%)
16	6	6	12082	1	1	27.3	27.5
15	24	30	12058	1	2	31.1	
14	111	141	11947	4	6	27.4	
13	525	666	11422	12	18	26.3	
12	1559	2225	9863	16	34	28.1	
11	880	3105	8983	31	65	28.8	
10	669	3774	8314	48	113	23.6	
9	1076	4850	7238	62	175	25.4	
8	639	5489	6599	49	224	22.2	
7	1545	7034	5054	60	284	18.9	
6	589	7623	4465	65	349	18.1	
5	881	8504	3584	51	400	20.1	
4	892	9396	2692	38	438	16.9	
3	384	9780	2308	15	453	19.7	
2	323	10103	1985	12	465	18.9	
1	29	10132	1956	2	467	18.7	

Table 87: VANTVEER

3.40 WANG-76

Hierachy Level	Genes removed	Cum. sum of genes removed	Genes left	GO-terms removed	Cum. sum of GO-terms removed	Acc. (%)	Sig.acc. (%)
17	6	6	12076	1	1	32.3	32.0
15	25	31	12051	3	4	34.4	
14	104	135	11947	7	11	32.0	
13	365	500	11582	12	23	28.6	
12	1649	2149	9933	27	50	30.9	
11	1048	3197	8885	38	88	30.3	
10	974	4171	7911	44	132	29.4	
9	1008	5179	6903	44	176	29.5	
8	1030	6209	5873	65	241	26.2	
7	1649	7858	4224	84	325	23.2	
6	643	8501	3581	62	387	25.2	
5	1001	9502	2580	46	433	26.8	
4	637	10139	1943	33	466	26.4	
3	295	10434	1648	19	485	24.8	
2	361	10795	1287	9	494	21.8	
1	17	10812	1270	3	497	19.1	

Table 88: WANG-76

3.41 WANG-ALK5T204D

Hierachy Level	Genes removed	Cum. sum of genes removed	Genes left	GO-terms removed	Cum. sum of GO-terms removed	Acc. (%)	Sig.acc. (%)
17	18	18	11946	2	2	72.8	73.6
16	43	61	11903	3	5	73.6	
15	76	137	11827	8	13	73.7	
14	239	376	11588	22	35	72.5	
13	687	1063	10901	35	70	70.6	
12	1790	2853	9111	57	127	71.4	
11	1254	4107	7857	83	210	69.3	
10	1151	5258	6706	98	308	71.1	
9	1465	6723	5241	146	454	74.4	
8	1189	7912	4052	159	613	69.8	
7	1219	9131	2833	148	761	64.0	
6	692	9823	2141	131	892	61.0	
5	609	10432	1532	86	978	57.2	
4	396	10828	1136	52	1030	61.8	
3	172	11000	964	26	1056	55.6	
2	233	11233	731	14	1070	28.7	
1	12	11245	719	3	1073	23.9	

Table 89: WANG-ALK5T204D

3.42 WELM

Hierachy Level	Genes removed	Cum. sum of genes removed	Genes left	GO-terms removed	Cum. sum of GO-terms removed	Acc. (%)	Sig.acc. (%)
12	54	54	12083	2	2	4.0	5.1
11	77	131	12006	2	4	3.8	
10	2	133	12004	1	5	4.1	
9	182	315	11822	3	8	3.5	
8	120	435	11702	4	12	5.6	
7	765	1200	10937	6	18	3.6	
6	370	1570	10567	3	21	4.9	
5	865	2435	9702	6	27	4.0	
4	1120	3555	8582	2	29	3.9	
3	32	3587	8550	1	30	4.6	
1	80	3667	8470	1	31	4.1	

Table 90: WELM

3.43 WEST

Hierachy Level	Genes removed	Cum. sum of genes removed	Genes left	GO-terms removed	Cum. sum of GO-terms removed	Acc. (%)	Sig.acc. (%)
16	68	68	11689	6	6	94.5	94.1
15	53	121	11636	9	15	93.4	
14	321	442	11315	34	49	92.8	
13	769	1211	10546	48	97	93.2	
12	1779	2990	8767	103	200	90.8	
11	1128	4118	7639	117	317	91.7	
10	1309	5427	6330	182	499	90.9	
9	1539	6966	4791	224	723	94.9	
8	1146	8112	3645	206	929	93.9	
7	1192	9304	2453	198	1127	92.5	
6	617	9921	1836	164	1291	91.9	
5	543	10464	1293	104	1395	96.0	
4	342	10806	951	66	1461	96.8	

Table 91: WEST

3.44 WHITFIELD

Hierachy Level	Genes removed	Cum. sum of genes removed	Genes left	GO-terms removed	Cum. sum of GO-terms removed	Acc. (%)	Sig.acc. (%)
16	18	18	11617	2	2	94.0	94.3
15	118	136	11499	15	17	95.2	
14	261	397	11238	31	48	94.9	
13	965	1362	10273	78	126	93.9	
12	1908	3270	8365	123	249	93.0	
11	1172	4442	7193	155	404	92.3	
10	1337	5779	5856	207	611	93.3	
9	1558	7337	4298	225	836	93.9	
8	1155	8492	3143	244	1080	91.9	
7	1005	9497	2138	211	1291	84.4	
6	621	10118	1517	181	1472	88.8	

Table 92: WHITFIELD

3.45 WONG-ESC

Hierachy Level	Genes removed	Cum. sum of genes removed	Genes left	GO-terms removed	Cum. sum of GO-terms removed	Acc. (%)	Sig.acc. (%)
16	30	30	11807	2	2	87.5	86.5
15	50	80	11757	7	9	87.9	
14	219	299	11538	20	29	87.2	
13	772	1071	10766	47	76	85.5	
12	1733	2804	9033	77	153	85.1	
11	1218	4022	7815	114	267	80.2	
10	1337	5359	6478	148	415	80.8	
9	1308	6667	5170	164	579	82.4	
8	941	7608	4229	145	724	74.7	
7	1171	8779	3058	157	881	69.2	
6	633	9412	2425	116	997	66.5	
5	812	10224	1613	82	1079	58.4	
4	458	10682	1155	50	1129	71.1	
3	216	10898	939	26	1155	46.5	
2	185	11083	754	14	1169	34.9	
1	14	11097	740	4	1173	26.2	

Table 93: WONG-ESC

3.46 WONG-MITOCHON

Hierachy Level	Genes removed	Cum. sum of genes removed	Genes left	GO-terms removed	Cum. sum of GO-terms removed	Acc. (%)	Sig.acc. (%)
18	17	17	11913	1	1	78.9	78.0
17	9	26	11904	3	4	74.9	
14	63	89	11841	9	13	76.7	
13	228	317	11613	12	25	78.0	
12	1228	1545	10385	25	50	78.7	
11	826	2371	9559	53	103	76.3	
10	1054	3425	8505	60	163	75.5	
9	1032	4457	7473	70	233	76.8	
8	949	5406	6524	77	310	78.6	
7	1754	7160	4770	89	399	69.7	
6	810	7970	3960	78	477	68.6	
5	868	8838	3092	67	544	63.4	
4	461	9299	2631	35	579	63.6	
3	339	9638	2292	24	603	65.6	
2	238	9876	2054	12	615	56.0	
1	30	9906	2024	3	618	56.6	

Table 94: WONG-MITOCHON

3.47 WONG-PROTEAS

Hierachy Level	Genes removed	Cum. sum of genes removed	Genes left	GO-terms removed	Cum. sum of GO-terms removed	Acc. (%)	Sig.acc. (%)
15	6	6	12090	1	1	25.7	28.5
14	33	39	12057	3	4	23.6	
13	317	356	11740	7	11	22.8	
12	1368	1724	10372	8	19	25.5	
11	874	2598	9498	18	37	23.2	
10	769	3367	8729	26	63	22.1	
9	838	4205	7891	22	85	24.6	
8	249	4454	7642	20	105	22.5	
7	1112	5566	6530	22	127	21.6	
6	264	5830	6266	20	147	20.1	
5	750	6580	5516	20	167	23.5	
4	785	7365	4731	13	180	22.0	
3	250	7615	4481	11	191	22.8	
2	140	7755	4341	3	194	19.4	
1	73	7828	4268	4	198	18.2	

Table 95: WONG-PROTEAS

3.48 YU

Hierachy Level	Genes removed	Cum. sum of genes removed	Genes left	GO-terms removed	Cum. sum of GO-terms removed	Acc. (%)	Sig.acc. (%)
16	32	32	12094	3	3	9.5	10.4
14	8	40	12086	2	5	10.8	
13	382	422	11704	7	12	12.6	
12	1396	1818	10308	12	24	9.9	
11	341	2159	9967	15	39	10.5	
10	186	2345	9781	24	63	10.7	
9	1108	3453	8673	34	97	10.1	
8	329	3782	8344	27	124	11.6	
7	1252	5034	7092	37	161	10.7	
6	478	5512	6614	39	200	11.8	
5	929	6441	5685	35	235	13.0	
4	626	7067	5059	22	257	11.3	
3	397	7464	4662	14	271	11.3	
2	447	7911	4215	5	276	10.3	
1	48	7959	4167	1	277	9.5	

Table 96: YU

4 GRP 2* with Bonferroni correction and removal of proliferation genes

Results for GRP 2*. The p-values are Bonferroni corrected.

4.1 ABBA

Hierachy Level	Genes removed	Cum. sum of genes removed	Genes left	GO-terms removed	Cum. sum of GO-terms removed	Acc. (%)
16	8	8	11520	2	2	40.4
15	13	21	11507	5	7	39.2
14	120	141	11387	12	19	37.3
13	438	579	10949	19	38	41.7
12	1706	2285	9243	44	82	40.1
11	1029	3314	8214	64	146	40.9
10	882	4196	7332	70	216	39.3
9	1204	5400	6128	95	311	37.7
8	906	6306	5222	101	412	37.1
7	1264	7570	3958	108	520	30.3
6	638	8208	3320	90	610	34.3
5	850	9058	2470	67	677	32.5
4	701	9759	1769	39	716	26.4
3	255	10014	1514	23	739	19.6
2	307	10321	1207	16	755	19.7
1	15	10336	1192	4	759	21.9

Table 97: ABBA

4.2 ADORNO

Hierachy Level	Genes removed	Cum. sum of genes removed	Genes left	GO-terms removed	Cum. sum of GO-terms removed	Acc. (%)
12	648	648	10916	2	2	1.5
11	243	891	10673	2	4	2.7
10	580	1471	10093	1	5	1.5
8	17	1488	10076	2	7	2.2
7	437	1925	9639	1	8	1.3
6	95	2020	9544	3	11	2.5
4	41	2061	9503	1	12	3.1
3	362	2423	9141	1	13	2.7
2	291	2714	8850	3	16	2.1
1	99	2813	8751	2	18	3.9

Table 98: ADORNO

4.3 BEN-PORATH-EXP1

Hierachy Level	Genes removed	Cum. sum of genes removed	Genes left	GO-terms removed	Cum. sum of GO-terms removed	Acc. (%)
18	17	17	11285	1	1	81.8
17	1	18	11284	1	2	81.7
16	39	57	11245	3	5	82.7
15	85	142	11160	12	17	83.8
14	282	424	10878	29	46	82.5
13	799	1223	10079	55	101	83.4
12	1751	2974	8328	94	195	79.5
11	1056	4030	7272	118	313	76.8
10	1246	5276	6026	176	489	75.2
9	1501	6777	4525	194	683	83.2
8	1052	7829	3473	185	868	84.2
7	1109	8938	2364	167	1035	81.8
6	639	9577	1725	148	1183	77.0
5	591	10168	1134	99	1282	72.9
4	344	10512	790	45	1327	87.9

Table 99: BEN-PORATH-EXP1

4.4 BEN-PORATH-PRC2

Hierachy Level	Genes removed	Cum. sum of genes removed	Genes left	GO-terms removed	Cum. sum of GO-terms removed	Acc. (%)
17	12	12	11109	4	4	89.7
16	59	71	11050	5	9	90.9
15	120	191	10930	20	29	89.3
14	273	464	10657	39	68	89.4
13	773	1237	9884	68	136	88.2
12	1554	2791	8330	108	244	89.5
11	929	3720	7401	135	379	85.6
10	1110	4830	6291	157	536	88.5
9	1386	6216	4905	218	754	93.5
8	1095	7311	3810	208	962	95.5
7	1073	8384	2737	177	1139	94.5
6	695	9079	2042	178	1317	94.4
5	566	9645	1476	113	1430	93.9

Table 100: BEN-PORATH-PRC2

4.5 BUESS

Hierachy Level	Genes removed	Cum. sum of genes removed	Genes left	GO-terms removed	Cum. sum of GO-terms removed	Acc. (%)
16	25	25	11520	1	1	10.8
14	3	28	11517	2	3	13.2
13	285	313	11232	10	13	13.6
12	1304	1617	9928	14	27	11.8
11	570	2187	9358	17	44	11.9
10	614	2801	8744	17	61	12.2
9	541	3342	8203	27	88	11.0
8	286	3628	7917	19	107	10.8
7	1415	5043	6502	36	143	11.0
6	560	5603	5942	29	172	10.7
5	707	6310	5235	24	196	9.7
4	606	6916	4629	16	212	10.4
3	238	7154	4391	5	217	10.5
2	44	7198	4347	3	220	11.3
1	82	7280	4265	2	222	9.3

Table 101: BUESS

4.6 BUFFA

Hierachy Level	Genes removed	Cum. sum of genes removed	Genes left	GO-terms removed	Cum. sum of GO-terms removed	Acc. (%)
16	24	24	11540	3	3	2.3
15	25	49	11515	2	5	2.2
14	26	75	11489	4	9	3.0
13	133	208	11356	5	14	3.2
12	1338	1546	10018	10	24	2.6
11	461	2007	9557	9	33	2.5
10	207	2214	9350	14	47	3.0
9	442	2656	8908	10	57	2.2
8	155	2811	8753	9	66	2.7
7	515	3326	8238	17	83	1.5
6	251	3577	7987	11	94	1.7
5	410	3987	7577	10	104	2.3
4	1058	5045	6519	7	111	3.0
3	319	5364	6200	3	114	3.1
2	1	5365	6199	1	115	2.5

Table 102: BUFFA

4.7 CARTER

Hierachy Level	Genes removed	Cum. sum of genes removed	Genes left	GO-terms removed	Cum. sum of GO-terms removed	Acc. (%)
16	5	5	11539	1	1	27.0
15	28	33	11511	2	3	29.5
14	54	87	11457	3	6	29.0
13	247	334	11210	9	15	30.6
12	1464	1798	9746	29	44	27.1
11	907	2705	8839	31	75	27.6
10	503	3208	8336	34	109	25.6
9	1299	4507	7037	61	170	26.2
8	507	5014	6530	53	223	25.5
7	1144	6158	5386	65	288	27.3
6	577	6735	4809	51	339	26.0
5	1050	7785	3759	36	375	27.3
4	542	8327	3217	26	401	25.3
3	456	8783	2761	23	424	25.5
2	309	9092	2452	10	434	20.2
1	107	9199	2345	4	438	18.6

Table 103: CARTER

4.8 CHANG

Hierachy Level	Genes removed	Cum. sum of genes removed	Genes left	GO-terms removed	Cum. sum of GO-terms removed	Acc. (%)
18	17	17	11273	1	1	77.9
17	11	28	11262	3	4	78.7
16	3	31	11259	2	6	78.3
15	85	116	11174	8	14	77.2
14	195	311	10979	24	38	76.9
13	753	1064	10226	40	78	76.7
12	1827	2891	8399	90	168	79.3
11	1154	4045	7245	111	279	74.2
10	1159	5204	6086	151	430	74.1
9	1254	6458	4832	169	599	73.1
8	1212	7670	3620	182	781	68.8
7	1214	8884	2406	188	969	63.0
6	689	9573	1717	162	1131	72.5
5	563	10136	1154	97	1228	63.6
4	333	10469	821	54	1282	76.9

Table 104: CHANG

4.9 CHI

Hierachy Level	Genes removed	Cum. sum of genes removed	Genes left	GO-terms removed	Cum. sum of GO-terms removed	Acc. (%)
16	20	20	11445	1	1	41.0
15	54	74	11391	4	5	40.5
14	187	261	11204	12	17	41.6
13	525	786	10679	17	34	38.3
12	1549	2335	9130	35	69	42.3
11	876	3211	8254	50	119	40.8
10	1030	4241	7224	65	184	42.6
9	1008	5249	6216	75	259	46.8
8	1038	6287	5178	95	354	46.5
7	1546	7833	3632	99	453	38.6
6	624	8457	3008	91	544	44.4
5	793	9250	2215	71	615	45.0
4	521	9771	1694	49	664	43.0
3	223	9994	1471	25	689	45.0
2	233	10227	1238	14	703	33.0
1	12	10239	1226	3	706	35.4

Table 105: CHI

4.10 CRAWFORD

Hierachy Level	Genes removed	Cum. sum of genes removed	Genes left	GO-terms removed	Cum. sum of GO-terms removed	Acc. (%)
16	26	26	11333	3	3	78.7
15	84	110	11249	10	13	79.6
14	207	317	11042	24	37	80.8
13	861	1178	10181	57	94	80.6
12	1768	2946	8413	85	179	79.8
11	1062	4008	7351	121	300	77.9
10	1230	5238	6121	162	462	76.5
9	1411	6649	4710	189	651	74.3
8	1105	7754	3605	187	838	66.2
7	1094	8848	2511	172	1010	70.9
6	651	9499	1860	149	1159	71.1
5	628	10127	1232	97	1256	61.4
4	369	10496	863	56	1312	71.3

Table 106: CRAWFORD

4.11 DAI

Hierachy Level	Genes removed	Cum. sum of genes removed	Genes left	GO-terms removed	Cum. sum of GO-terms removed	Acc. (%)
16	5	5	11551	1	1	15.6
14	9	14	11542	1	2	15.8
13	127	141	11415	5	7	17.0
12	1308	1449	10107	9	16	15.0
11	836	2285	9271	23	39	17.1
10	562	2847	8709	19	58	16.3
9	918	3765	7791	34	92	17.5
8	405	4170	7386	24	116	14.5
7	1082	5252	6304	42	158	13.4
6	750	6002	5554	33	191	14.4
5	708	6710	4846	20	211	13.1
4	1209	7919	3637	14	225	16.3
3	296	8215	3341	11	236	15.1
2	419	8634	2922	7	243	12.7
1	37	8671	2885	2	245	10.3

Table 107: DAI

4.12 GLINSKY

Hierachy Level	Genes removed	Cum. sum of genes removed	Genes left	GO-terms removed	Cum. sum of GO-terms removed	Acc. (%)
16	5	5	11555	1	1	5.9
15	5	10	11550	1	2	5.8
14	17	27	11533	2	4	5.5
13	231	258	11302	6	10	7.0
12	1423	1681	9879	12	22	5.2
11	352	2033	9527	17	39	5.9
10	589	2622	8938	17	56	6.1
9	622	3244	8316	18	74	7.0
8	184	3428	8132	16	90	7.0
7	645	4073	7487	26	116	5.4
6	489	4562	6998	26	142	6.8
5	813	5375	6185	21	163	6.0
4	1114	6489	5071	17	180	6.1
3	131	6620	4940	5	185	5.3
2	88	6708	4852	3	188	5.5
1	24	6732	4828	1	189	5.2

Table 108: GLINSKY

4.13 HALLSTROM

Hierachy Level	Genes removed	Cum. sum of genes removed	Genes left	GO-terms removed	Cum. sum of GO-terms removed	Acc. (%)
15	64	64	11462	3	3	28.1
14	51	115	11411	4	7	27.7
13	421	536	10990	12	19	27.5
12	1460	1996	9530	24	43	27.7
11	764	2760	8766	31	74	26.0
10	876	3636	7890	51	125	25.5
9	1135	4771	6755	53	178	29.5
8	797	5568	5958	66	244	28.9
7	1629	7197	4329	74	318	28.1
6	576	7773	3753	65	383	31.2
5	1007	8780	2746	48	431	35.0
4	568	9348	2178	25	456	33.3
3	216	9564	1962	16	472	32.0
2	310	9874	1652	9	481	29.2
1	21	9895	1631	4	485	26.7

Table 109: HALLSTROM

4.14 HE

Hierachy Level	Genes removed	Cum. sum of genes removed	Genes left	GO-terms removed	Cum. sum of GO-terms removed	Acc. (%)
15	6	6	11554	1	1	5.6
12	885	891	10669	1	2	3.5
11	86	977	10583	3	5	5.4
10	122	1099	10461	6	11	4.5
9	511	1610	9950	10	21	3.5
8	132	1742	9818	8	29	4.4
7	878	2620	8940	7	36	5.9
6	402	3022	8538	8	44	4.7
5	461	3483	8077	4	48	3.8
4	1663	5146	6414	7	55	5.1
3	352	5498	6062	3	58	3.5
1	22	5520	6040	1	59	4.0

Table 110: HE

4.15 HU

Hierachy Level	Genes removed	Cum. sum of genes removed	Genes left	GO-terms removed	Cum. sum of GO-terms removed	Acc. (%)
14	21	21	11535	2	2	5.9
13	312	333	11223	3	5	6.8
12	800	1133	10423	4	9	7.5
11	260	1393	10163	6	15	6.0
10	720	2113	9443	6	21	7.5
9	111	2224	9332	3	24	7.8
8	431	2655	8901	14	38	6.2
7	805	3460	8096	14	52	7.3
6	170	3630	7926	10	62	4.3
5	787	4417	7139	9	71	6.6
4	145	4562	6994	5	76	6.4
3	548	5110	6446	4	80	7.1
2	333	5443	6113	1	81	6.3

Table 111: HU

4.16 HUA

Hierachy Level	Genes removed	Cum. sum of genes removed	Genes left	GO-terms removed	Cum. sum of GO-terms removed	Acc. (%)
17	36	36	10493	7	7	97.3
16	65	101	10428	11	18	98.2
15	144	245	10284	20	38	99.3
14	391	636	9893	74	112	96.9
13	892	1528	9001	120	232	97.3
12	1719	3247	7282	208	440	95.3
11	1197	4444	6085	239	679	96.1
10	1348	5792	4737	302	981	94.9
9	1443	7235	3294	325	1306	97.4

Table 112: HUA

4.17 IVSHINA

Hierachy Level	Genes removed	Cum. sum of genes removed	Genes left	GO-terms removed	Cum. sum of GO-terms removed	Acc. (%)
14	10	10	11551	1	1	7.3
13	59	69	11492	2	3	7.6
12	1273	1342	10219	7	10	8.2
11	638	1980	9581	7	17	8.0
10	353	2333	9228	7	24	8.1
9	529	2862	8699	16	40	7.3
8	258	3120	8441	25	65	7.5
7	687	3807	7754	20	85	9.1
6	337	4144	7417	15	100	6.2
5	671	4815	6746	12	112	7.4
4	420	5235	6326	13	125	6.2
3	497	5732	5829	11	136	5.7
2	97	5829	5732	6	142	6.3
1	32	5861	5700	1	143	5.8

Table 113: IVSHINA

4.18 KOK

Hierachy Level	Genes removed	Cum. sum of genes removed	Genes left	GO-terms removed	Cum. sum of GO-terms removed	Acc. (%)
116	31	31	11417	2	2	52.8
15	84	115	11333	7	9	55.7
14	150	265	11183	12	21	52.7
13	638	903	10545	25	46	52.0
12	1609	2512	8936	52	98	53.9
11	1092	3604	7844	64	162	52.8
10	959	4563	6885	78	240	53.8
9	1333	5896	5552	106	346	60.4
8	1115	7011	4437	124	470	59.9
7	1235	8246	3202	115	585	52.9
6	694	8940	2508	107	692	59.5
5	798	9738	1710	78	770	62.2
4	463	10201	1247	51	821	54.8
3	198	10399	1049	32	853	55.0
2	200	10599	849	13	866	45.9
1	14	10613	835	3	869	42.1

Table 114: KOK

4.19 KORKOLA

Hierachy Level	Genes removed	Cum. sum of genes removed	Genes left	GO-terms removed	Cum. sum of GO-terms removed	Acc. (%)
16	23	23	11526	2	2	10.3
15	5	28	11521	1	3	9.3
14	43	71	11478	4	7	10.7
13	343	414	11135	8	15	10.0
12	1309	1723	9826	15	30	9.6
11	616	2339	9210	17	47	9.1
10	873	3212	8337	34	81	10.6
9	855	4067	7482	33	114	12.0
8	420	4487	7062	33	147	9.6
7	980	5467	6082	27	174	11.1
6	743	6210	5339	38	212	10.1
5	1012	7222	4327	28	240	12.6
4	856	8078	3471	24	264	11.1
3	311	8389	3160	13	277	10.8
2	442	8831	2718	9	286	8.6
1	66	8897	2652	2	288	7.1

Table 115: KORKOLA

4.20 LIU

Hierachy Level	Genes removed	Cum. sum of genes removed	Genes left	GO-terms removed	Cum. sum of GO-terms removed	Acc. (%)
15	93	93	11350	4	4	42.7
14	119	212	11231	12	16	49.6
13	520	732	10711	29	45	45.7
12	1746	2478	8965	54	99	46.6
11	1117	3595	7848	81	180	46.5
10	1227	4822	6621	89	269	46.9
9	1177	5999	5444	107	376	48.9
8	828	6827	4616	94	470	49.9
7	1372	8199	3244	112	582	40.6
6	640	8839	2604	108	690	39.0
5	751	9590	1853	84	774	45.8
4	533	10123	1320	44	818	40.9
3	207	10330	1113	29	847	39.3
2	219	10549	894	14	861	20.0
1	8	10557	886	4	865	20.2

Table 116: LIU

4.21 MA

Hierachy Level	Genes removed	Cum. sum of genes removed	Genes left	GO-terms removed	Cum. sum of GO-terms removed	Acc. (%)
15	17	17	11542	1	1	13.4
14	30	47	11512	4	5	14.0
13	277	324	11235	6	11	13.3
12	1343	1667	9892	16	27	15.5
11	631	2298	9261	25	52	14.3
10	372	2670	8889	23	75	14.2
9	1030	3700	7859	32	107	14.7
8	371	4071	7488	29	136	13.0
7	952	5023	6536	41	177	11.0
6	720	5743	5816	29	206	13.6
5	771	6514	5045	18	224	12.1
4	1140	7654	3905	13	237	11.3
3	306	7960	3599	8	245	12.7
2	430	8390	3169	6	251	8.9
1	80	8470	3089	3	254	9.6

Table 117: MA

4.22 MILLER

Hierachy Level	Genes removed	Cum. sum of genes removed	Genes left	GO-terms removed	Cum. sum of GO-terms removed	Acc. (%)
14	33	33	11519	1	1	8.8
12	1306	1339	10213	6	7	9.8
11	607	1946	9606	13	20	10.4
10	322	2268	9284	12	32	9.7
9	196	2464	9088	14	46	7.4
8	292	2756	8796	16	62	9.2
7	605	3361	8191	16	78	8.1
6	544	3905	7647	22	100	9.4
5	875	4780	6772	14	114	6.8
4	1164	5944	5608	11	125	8.9
3	801	6745	4807	11	136	7.8
2	397	7142	4410	4	140	7.5
1	157	7299	4253	2	142	7.2

Table 118: MILLER

4.23 MORI

Hierachy Level	Genes removed	Cum. sum of genes removed	Genes left	GO-terms removed	Cum. sum of GO-terms removed	Acc. (%)
16	24	24	11432	2	2	44.8
15	72	96	11360	8	10	45.2
14	152	248	11208	12	22	44.8
13	536	784	10672	17	39	47.7
12	1769	2553	8903	41	80	47.1
11	994	3547	7909	50	130	47.1
10	1036	4583	6873	75	205	47.4
9	1159	5742	5714	87	292	47.8
8	929	6671	4785	90	382	48.1
7	1270	7941	3515	80	462	45.0
6	691	8632	2824	91	553	42.9
5	778	9410	2046	56	609	46.4
4	549	9959	1497	38	647	45.3
3	247	10206	1250	25	672	36.4
2	263	10469	987	14	686	32.1
1	15	10484	972	4	690	26.8

Table 119: MORI

4.24 PAIK

Hierachy Level	Genes removed	Cum. sum of genes removed	Genes left	GO-terms removed	Cum. sum of GO-terms removed	Acc. (%)
15	5	5	11549	1	1	8.8
14	72	77	11477	5	6	9.1
13	217	294	11260	4	10	8.3
12	1417	1711	9843	19	29	8.6
11	788	2499	9055	22	51	7.7
10	443	2942	8612	15	66	5.8
9	978	3920	7634	35	101	9.5
8	317	4237	7317	25	126	9.4
7	792	5029	6525	43	169	8.1
6	785	5814	5740	53	222	7.9
5	845	6659	4895	36	258	8.1
4	986	7645	3909	22	280	8.6
3	325	7970	3584	20	300	8.0
2	77	8047	3507	9	309	5.8
1	16	8063	3491	2	311	7.3

Table 120: PAIK

4.25 PAWITAN

Hierachy Level	Genes removed	Cum. sum of genes removed	Genes left	GO-terms removed	Cum. sum of GO-terms removed	Acc. (%)
17	1	1	11543	1	1	17.2
15	11	12	11532	1	2	19.1
14	127	139	11405	11	13	17.1
13	287	426	11118	7	20	17.9
12	1465	1891	9653	21	41	18.7
11	726	2617	8927	32	73	17.9
10	737	3354	8190	46	119	20.0
9	606	3960	7584	45	164	17.3
8	892	4852	6692	63	227	17.7
7	1527	6379	5165	68	295	16.3
6	437	6816	4728	54	349	15.9
5	1073	7889	3655	47	396	18.5
4	622	8511	3033	26	422	16.6
3	212	8723	2821	16	438	15.6
2	450	9173	2371	10	448	13.2
1	11	9184	2360	2	450	11.2

Table 121: PAWITAN

4.26 PEI

Hierachy Level	Genes removed	Cum. sum of genes removed	Genes left	GO-terms removed	Cum. sum of GO-terms removed	Acc. (%)
16	3	3	11562	1	1	2.9
15	7	10	11555	3	4	3.7
14	2	12	11553	1	5	2.8
13	171	183	11382	2	7	3.5
12	1236	1419	10146	9	16	2.2
11	565	1984	9581	8	24	2.7
10	412	2396	9169	10	34	2.9
9	216	2612	8953	11	45	1.2
8	175	2787	8778	13	58	2.5
7	191	2978	8587	9	67	2.2
6	497	3475	8090	24	91	3.1
5	826	4301	7264	16	107	2.4
4	816	5117	6448	4	111	2.4
3	122	5239	6326	4	115	2.3
2	202	5441	6124	3	118	1.5
1	49	5490	6075	1	119	3.0

Table 122: PEI

4.27 RAMASWAMY

Hierachy Level	Genes removed	Cum. sum of genes removed	Genes left	GO-terms removed	Cum. sum of GO-terms removed	Acc. (%)
15	17	17	11538	1	1	6.7
14	7	24	11531	3	4	8.6
13	49	73	11482	1	5	8.0
12	1556	1629	9926	13	18	5.5
11	710	2339	9216	9	27	7.1
10	564	2903	8652	19	46	8.1
9	244	3147	8408	8	54	7.9
8	457	3604	7951	27	81	8.2
7	1691	5295	6260	33	114	7.0
6	532	5827	5728	37	151	6.7
5	498	6325	5230	23	174	6.5
4	884	7209	4346	18	192	6.1
3	85	7294	4261	4	196	7.0
2	175	7469	4086	6	202	7.2
1	80	7549	4006	1	203	7.4

Table 123: RAMASWAMY

4.28 REUTER

Hierachy Level	Genes removed	Cum. sum of genes removed	Genes left	GO-terms removed	Cum. sum of GO-terms removed	Acc. (%)
17	14	14	10981	5	5	96.2
16	79	93	10902	6	11	95.4
15	141	234	10761	21	32	95.5
14	287	521	10474	42	74	94.4
13	832	1353	9642	68	142	95.4
12	1788	3141	7854	135	277	93.0
11	1172	4313	6682	164	441	92.6
10	1271	5584	5411	215	656	94.4
9	1459	7043	3952	248	904	97.9
8	1138	8181	2814	272	1176	99.2
7	982	9163	1832	218	1394	98.4

Table 124: REUTER

4.29 RHODES

Hierachy Level	Genes removed	Cum. sum of genes removed	Genes left	GO-terms removed	Cum. sum of GO-terms removed	Acc. (%)
16	30	30	11508	2	2	25.5
15	5	35	11503	1	3	23.9
14	10	45	11493	2	5	25.4
13	231	276	11262	9	14	26.5
12	1557	1833	9705	23	37	25.6
11	1041	2874	8664	46	83	25.9
10	752	3626	7912	49	132	26.3
9	1208	4834	6704	66	198	24.7
8	964	5798	5740	72	270	23.6
7	1206	7004	4534	75	345	23.0
6	717	7721	3817	68	413	22.3
5	941	8662	2876	50	463	24.1
4	639	9301	2237	25	488	21.4
3	376	9677	1861	20	508	19.0
2	379	10056	1482	12	520	20.2
1	25	10081	1457	4	524	16.1

Table 125: RHODES

4.30 SAAL

Hierachy Level	Genes removed	Cum. sum of genes removed	Genes left	GO-terms removed	Cum. sum of GO-terms removed	Acc. (%)
18	17	17	11438	1	1	50.5
17	8	25	11430	2	3	48.6
16	30	55	11400	4	7	50.4
15	23	78	11377	5	12	50.5
14	245	323	11132	19	31	49.8
13	672	995	10460	33	64	46.2
12	1814	2809	8646	65	129	48.0
11	967	3776	7679	64	193	48.3
10	1027	4803	6652	78	271	48.5
9	1065	5868	5587	107	378	49.4
8	975	6843	4612	128	506	52.2
7	1182	8025	3430	121	627	42.5
6	673	8698	2757	110	737	42.3
5	769	9467	1988	76	813	41.6
4	525	9992	1463	46	859	38.7
3	249	10241	1214	28	887	33.4
2	258	10499	956	12	899	21.7
1	23	10522	933	3	902	16.9

Table 126: SAAL

4.31 SHIPITSIN

Hierachy Level	Genes removed	Cum. sum of genes removed	Genes left	GO-terms removed	Cum. sum of GO-terms removed	Acc. (%)
16	24	24	11492	2	2	26.3
15	37	61	11455	4	6	21.2
14	89	150	11366	8	14	23.4
13	394	544	10972	15	29	22.2
12	1608	2152	9364	33	62	23.0
11	868	3020	8496	42	104	20.7
10	859	3879	7637	45	149	24.6
9	1039	4918	6598	63	212	23.4
8	1056	5974	5542	74	286	25.4
7	1367	7341	4175	69	355	23.5
6	615	7956	3560	73	428	23.2
5	810	8766	2750	47	475	25.1
4	590	9356	2160	36	511	24.7
3	273	9629	1887	13	524	21.1
2	371	10000	1516	9	533	18.5
1	21	10021	1495	5	538	16.1

Table 127: SHIPITSIN

4.32 SORLIE

Hierachy Level	Genes removed	Cum. sum of genes removed	Genes left	GO-terms removed	Cum. sum of GO-terms removed	Acc. (%)
16	25	25	11528	1	1	7.5
13	177	202	11351	2	3	8.7
12	1436	1638	9915	7	10	7.7
11	221	1859	9694	7	17	8.7
10	743	2602	8951	13	30	9.0
9	527	3129	8424	12	42	7.8
8	333	3462	8091	19	61	7.3
7	855	4317	7236	25	86	10.3
6	569	4886	6667	22	108	8.6
5	1089	5975	5578	22	130	8.1
4	599	6574	4979	14	144	10.5
3	368	6942	4611	9	153	8.2
2	109	7051	4502	3	156	9.3
1	22	7073	4480	2	158	8.4

Table 128: SORLIE

4.33 SOTIRIOU-93

Hierachy Level	Genes removed	Cum. sum of genes removed	Genes left	GO-terms removed	Cum. sum of GO-terms removed	Acc. (%)
16	72	72	11219	9	9	80.1
15	108	180	11111	12	21	78.4
14	256	436	10855	28	49	78.8
13	784	1220	10071	67	116	78.2
12	1751	2971	8320	111	227	77.1
11	1118	4089	7202	116	343	73.9
10	1353	5442	5849	171	514	78.2
9	1436	6878	4413	217	731	75.1
8	1254	8132	3159	256	987	75.2
7	988	9120	2171	195	1182	74.0
6	613	9733	1558	173	1355	74.6
5	503	10236	1055	93	1448	77.2
4	299	10535	756	57	1505	78.2

Table 129: SOTIRIOU-93

4.34 SOTIRIOU-GGI

Hierachy Level	Genes removed	Cum. sum of genes removed	Genes left	GO-terms removed	Cum. sum of GO-terms removed	Acc. (%)
16	5	5	11530	1	1	33.5
15	5	10	11525	1	2	32.6
14	27	37	11498	4	6	34.2
13	407	444	11091	6	12	32.2
12	1519	1963	9572	27	39	35.4
11	979	2942	8593	36	75	32.8
10	645	3587	7948	45	120	34.0
9	777	4364	7171	50	170	34.8
8	725	5089	6446	69	239	33.7
7	1659	6748	4787	83	322	28.6
6	752	7500	4035	64	386	36.4
5	830	8330	3205	37	423	35.3
4	783	9113	2422	28	451	33.0
3	195	9308	2227	24	475	32.4
2	495	9803	1732	15	490	28.7
1	24	9827	1708	2	492	28.5

Table 130: SOTIRIOU-GGI

4.35 META-PCNA

Hierachy Level	Genes removed	Cum. sum of genes removed	Genes left	GO-terms removed	Cum. sum of GO-terms removed	Acc. (%)
16	5	5	11561	1	1	45.3
15	30	35	11531	3	4	42.0
14	13	48	11518	2	6	45.0
13	481	529	11037	11	17	42.1
12	1509	2038	9528	34	51	43.7
11	981	3019	8547	52	103	44.4
10	932	3951	7615	58	161	43.8
9	1201	5152	6414	72	233	43.9
8	556	5708	5858	66	299	45.6
7	1369	7077	4489	95	394	40.3
6	738	7815	3751	82	476	43.0
5	816	8631	2935	57	533	44.6
4	798	9429	2137	35	568	44.8
3	411	9840	1726	25	593	38.8
2	338	10178	1388	17	610	34.9
1	7	10185	1381	3	613	32.5

Table 131: META-PCNA

4.36 TAUBE

Hierachy Level	Genes removed	Cum. sum of genes removed	Genes left	GO-terms removed	Cum. sum of GO-terms removed	Acc. (%)
17	6	6	11360	2	2	66.8
16	66	72	11294	7	9	66.5
15	77	149	11217	9	18	66.3
14	167	316	11050	24	42	65.2
13	608	924	10442	38	80	64.7
12	1642	2566	8800	67	147	69.6
11	863	3429	7937	76	223	63.2
10	1055	4484	6882	107	330	60.8
9	1368	5852	5514	150	480	71.4
8	1095	6947	4419	155	635	70.8
7	1166	8113	3253	135	770	68.6
6	675	8788	2578	146	916	64.3
5	653	9441	1925	94	1010	67.2
4	451	9892	1474	54	1064	62.8
3	178	10070	1296	26	1090	49.3
2	198	10268	1098	11	1101	30.7
1	21	10289	1077	4	1105	32.8

Table 132: TAUBE

4.37 TAVAZOIE

Hierachy Level	Genes removed	Cum. sum of genes removed	Genes left	GO-terms removed	Cum. sum of GO-terms removed	Acc. (%)
14	45	45	11516	1	1	4.1
13	107	152	11409	3	4	4.9
12	856	1008	10553	2	6	3.7
11	507	1515	10046	5	11	4.4
10	634	2149	9412	14	25	3.7
9	546	2695	8866	13	38	4.5
8	215	2910	8651	16	54	4.4
7	1492	4402	7159	24	78	3.9
6	695	5097	6464	32	110	3.5
5	1042	6139	5422	26	136	3.7
4	540	6679	4882	15	151	4.1
3	346	7025	4536	7	158	4.1
2	124	7149	4412	2	160	4.7

Table 133: TAVAZOIE

4.38 VALASTYAN

Hierachy Level	Genes removed	Cum. sum of genes removed	Genes left	GO-terms removed	Cum. sum of GO-terms removed	Acc. (%)
14	68	68	11493	7	7	3.6
13	39	107	11454	4	11	4.6
12	160	267	11294	6	17	5.3
11	853	1120	10441	9	26	6.8
10	303	1423	10138	13	39	6.4
9	723	2146	9415	19	58	4.3
8	632	2778	8783	32	90	2.5
7	444	3222	8339	16	106	4.3
6	400	3622	7939	22	128	5.0
5	1096	4718	6843	22	150	4.7
4	650	5368	6193	16	166	5.4
3	385	5753	5808	7	173	5.7
2	300	6053	5508	5	178	3.4

Table 134: VALASTYAN

4.39 VANTVEER

Hierachy Level	Genes removed	Cum. sum of genes removed	Genes left	GO-terms removed	Cum. sum of GO-terms removed	Acc. (%)
16	5	5	11524	1	1	20.1
15	23	28	11501	1	2	21.2
14	96	124	11405	4	6	22.1
13	486	610	10919	12	18	21.8
12	1481	2091	9438	15	33	22.1
11	810	2901	8628	30	63	22.2
10	637	3538	7991	48	111	21.3
9	1010	4548	6981	60	171	21.9
8	613	5161	6368	48	219	19.8
7	1462	6623	4906	60	279	18.7
6	570	7193	4336	64	343	18.0
5	866	8059	3470	51	394	19.0
4	867	8926	2603	37	431	16.5
3	379	9305	2224	15	446	16.2
2	303	9608	1921	12	458	17.7
1	29	9637	1892	2	460	13.3

Table 135: VANTVEER

4.40 WANG-76

Hierachy Level	Genes removed	Cum. sum of genes removed	Genes left	GO-terms removed	Cum. sum of GO-terms removed	Acc. (%)
17	6	6	11516	1	1	25.3
15	22	28	11494	3	4	25.7
14	89	117	11405	7	11	26.7
13	333	450	11072	12	23	23.9
12	1558	2008	9514	27	50	22.9
11	955	2963	8559	37	87	25.5
10	931	3894	7628	44	131	25.9
9	942	4836	6686	43	174	26.6
8	978	5814	5708	65	239	22.1
7	1589	7403	4119	84	323	23.1
6	625	8028	3494	61	384	25.6
5	980	9008	2514	45	429	25.2
4	621	9629	1893	33	462	25.3
3	290	9919	1603	19	481	20.4
2	350	10269	1253	9	490	19.1
1	17	10286	1236	3	493	19.0

Table 136: WANG-76

4.41 WANG-ALK5T204D

Hierachy Level	Genes removed	Cum. sum of genes removed	Genes left	GO-terms removed	Cum. sum of GO-terms removed	Acc. (%)
17	18	18	11378	2	2	59.0
16	41	59	11337	3	5	60.3
15	72	131	11265	8	13	62.3
14	228	359	11037	21	34	60.0
13	656	1015	10381	35	69	61.7
12	1691	2706	8690	56	125	60.4
11	1149	3855	7541	82	207	57.8
10	1098	4953	6443	98	305	58.8
9	1392	6345	5051	143	448	59.4
8	1127	7472	3924	159	607	56.6
7	1175	8647	2749	146	753	56.7
6	672	9319	2077	130	883	54.1
5	594	9913	1483	86	969	52.0
4	385	10298	1098	52	1021	49.8
3	169	10467	929	25	1046	38.4
2	216	10683	713	13	1059	22.6
1	12	10695	701	3	1062	18.0

Table 137: WANG-ALK5T204D

4.42 WELM

Hierachy Level	Genes removed	Cum. sum of genes removed	Genes left	GO-terms removed	Cum. sum of GO-terms removed	Acc. (%)
12	49	49	11514	2	2	3.1
11	74	123	11440	2	4	3.6
10	2	125	11438	1	5	3.9
9	175	300	11263	3	8	3.3
8	117	417	11146	4	12	2.5
7	715	1132	10431	6	18	3.6
6	351	1483	10080	3	21	2.8
5	828	2311	9252	6	27	3.1
4	1090	3401	8162	2	29	3.0
3	31	3432	8131	1	30	3.2
1	74	3506	8057	1	31	4.1

Table 138: WELM

4.43 WEST

Hierachy Level	Genes removed	Cum. sum of genes removed	Genes left	GO-terms removed	Cum. sum of GO-terms removed	Acc. (%)
16	66	66	11139	6	6	84.5
15	50	116	11089	9	15	83.9
14	298	414	10791	34	49	84.4
13	723	1137	10068	48	97	84.3
12	1669	2806	8399	103	200	82.0
11	1066	3872	7333	117	317	83.4
10	1259	5131	6074	179	496	82.0
9	1450	6581	4624	223	719	88.9
8	1109	7690	3515	204	923	89.5
7	1143	8833	2372	197	1120	85.6
6	599	9432	1773	164	1284	83.8
5	530	9962	1243	103	1387	90.2

Table 139: WEST

4.44 WHITFIELD

Hierachy Level	Genes removed	Cum. sum of genes removed	Genes left	GO-terms removed	Cum. sum of GO-terms removed	Acc. (%)
16	18	18	11548	2	2	93.7
15	116	134	11432	14	16	92.7
14	261	395	11171	31	47	93.1
13	955	1350	10216	78	125	92.9
12	1897	3247	8319	122	247	90.4
11	1160	4407	7159	153	400	89.1
10	1328	5735	5831	206	606	90.2
9	1546	7281	4285	225	831	93.8
8	1150	8431	3135	244	1075	90.1
7	1005	9436	2130	211	1286	85.4
6	619	10055	1511	181	1467	87.3

Table 140: WHITFIELD

4.45 WONG-ESC

Hierachy Level	Genes removed	Cum. sum of genes removed	Genes left	GO-terms removed	Cum. sum of GO-terms removed	Acc. (%)
16	30	30	11305	2	2	78.9
15	46	76	11259	6	8	79.3
14	209	285	11050	20	28	79.2
13	712	997	10338	46	74	78.2
12	1641	2638	8697	76	150	77.5
11	1139	3777	7558	110	260	77.7
10	1278	5055	6280	145	405	72.0
9	1250	6305	5030	159	564	74.3
8	900	7205	4130	142	706	70.2
7	1139	8344	2991	153	859	67.2
6	622	8966	2369	114	973	63.3
5	797	9763	1572	81	1054	55.9
4	449	10212	1123	50	1104	63.0
3	212	10424	911	26	1130	38.1
2	177	10601	734	14	1144	28.5
1	14	10615	720	4	1148	23.1

Table 141: WONG-ESC

4.46 WONG-MITOCHON

Hierachy Level	Genes removed	Cum. sum of genes removed	Genes left	GO-terms removed	Cum. sum of GO-terms removed	Acc. (%)
18	17	17	11344	1	1	64.3
17	9	26	11335	3	4	64.7
14	62	88	11273	9	13	64.1
13	214	302	11059	12	25	62.9
12	1154	1456	9905	25	50	62.2
11	784	2240	9121	51	101	63.8
10	1012	3252	8109	59	160	63.2
9	976	4228	7133	69	229	63.2
8	901	5129	6232	77	306	59.9
7	1632	6761	4600	89	395	59.2
6	789	7550	3811	78	473	52.0
5	829	8379	2982	66	539	51.9
4	446	8825	2536	34	573	51.4
3	327	9152	2209	24	597	49.2
2	233	9385	1976	12	609	42.6
1	27	9412	1949	3	612	40.8

Table 142: WONG-MITOCHON

4.47 WONG-PROTEAS

Hierachy Level	Genes removed	Cum. sum of genes removed	Genes left	GO-terms removed	Cum. sum of GO-terms removed	Acc. (%)
15	6	6	11521	1	1	20.1
14	22	28	11499	3	4	16.6
13	300	328	11199	7	11	18.0
12	1277	1605	9922	8	19	20.5
11	814	2419	9108	18	37	18.4
10	740	3159	8368	26	63	18.2
9	769	3928	7599	22	85	20.7
8	235	4163	7364	20	105	20.3
7	1063	5226	6301	22	127	17.0
6	246	5472	6055	20	147	18.7
5	723	6195	5332	20	167	18.1
4	760	6955	4572	13	180	16.8
3	242	7197	4330	11	191	16.9
2	115	7312	4215	3	194	19.2
1	68	7380	4147	4	198	18.2

Table 143: WONG-PROTEAS

4.48 YU

Hierachy Level	Genes removed	Cum. sum of genes removed	Genes left	GO-terms removed	Cum. sum of GO-terms removed	Acc. (%)
16	32	32	11520	3	3	8.3
14	7	39	11513	2	5	9.2
13	370	409	11143	7	12	8.4
12	1310	1719	9833	12	24	7.2
11	324	2043	9509	15	39	6.9
10	181	2224	9328	24	63	8.0
9	1053	3277	8275	34	97	9.1
8	311	3588	7964	27	124	6.4
7	1199	4787	6765	37	161	8.4
6	453	5240	6312	38	199	8.4
5	897	6137	5415	35	234	6.3
4	591	6728	4824	21	255	8.9
3	380	7108	4444	14	269	8.7
2	433	7541	4011	5	274	8.5
1	46	7587	3965	1	275	8.5

Table 144: YU

5 Overlap of genes and GO-terms

At the lowest hierarchy level, many of the signatures have an accuracy below 15%. This is with the exception of a few signatures namely, Ben-porath-exp1, Ben-porath-prc2, Chang, Chi, Crawford, Hua, Kok, Reuter, Sotiriou-93, Taube, West, Whitfield and Wong-mitochon. And among these reported signatures, only Ben-porath-exp1, Ben-porath-prc2, Chang, Hua, Reuter, Sotiriou-93 and West have an accuracy greater than 50%. Furthermore, only four signatures have an accuracy at the lowest level at 90% or higher. These four signatures are Hua, Ben-porath-prc2, Reuter and West. They are among the five largest gene signatures we have. All four signatures have gene count greater than 460. Also, the smallest signatures - Pei, Adorno, Buffa and Welm also have the lowest accuracy at the last level. The percentage accuracy of these four smallest signatures are 0.8%, 1.2%, 1.7% and 2.1% respectively. Therefore, we can say that the higher the number of genes in a signature the more likely that the accuracy will be higher.

Next, we study the gene similarity between the four signatures: Hua, Ben-porath-prc2, Reuter and West. To explain this, we found the number of remaining genes at the last level for each of these four signatures. There are 3397, 1536, 1887 and 951 genes remaining at the last level for the signatures Hua, Ben-porath-prc2, Reuter and West respectively. For studying the intersections of these sets we constructed a Venn diagram. The overlap of these sets is shown in Figure 1 A. From this figure, one can see that all four signatures them have 433 genes in common.

After finding the gene overlaps between the four signatures, we also examined the GO-terms of BP that are common to all four of them. The remaining genes in each signature are mapped to GO-terms of BP. That is to say for the 3397 remaining genes for the study Hua, we mapped the genes to GO-terms. This was also done for all the four signatures and the intersection was derived. The resulting intersections among the sets of GO-terms is shown in the Venn diagram in Figure 1 B. In total there are 344 GO-terms common in all four signatures.

In order to study if the remaining 344 GO-terms from these intersections capture relevant predictive information, we identify all genes associated with these GO-terms. From this mapping we find 2231 genes. From this we form a new gene set by removing these 2231 genes from the breast cancer dataset. This generates a new gene set G_r. For G_r we determine now the overlap with each of our 48 biomarker sets. Hence, this analysis allows us to identify the overlap between the original biomarker sets and G_r. Specifically, the overlap in percentage is measures by

$$p_{bm}(i) = \frac{|G_r \cap BM_i|}{|BM_i|}, \tag{1}$$

whereas BM_i is biomarker set i. The results for each biomarker is shown in Table 145. All the signatures have at least 60% of their genes present in G_r. The signatures with a value of 1 in Table 145 are those that are smaller in size. From this one can conclude that

5.1 Gene signatures overlap with the 433 genes

We also analyzed the overlap between the 48 gene signatures and the 433 genes specific to the four signatures that were discussed earlier. The results are presented in the third column of Table 145. It is noted that most of the biomarkers do not overlap with the 433 genes. To put it simply, there are 27 gene signatures that have no intersection with the 433 genes. This means only 21 biomarkers have an overlap with the genes and even then the value of the overlap is insignificant for some of them. For such 21 biomarkers, 6 of them have only one gene overlap. Only two signatures have an overlap that surpass 10 genes. These signatures are Whitfield and Wong-mitochon.

5.2 Comparison between the signatures

During the study we found 4 biomarkers set that after all hierarchy levels had an accuracy at 90% or higher (this is the level with the most genes removed). We also do have 44 gene signatures whose accurary is less than 90%, therefore for the two sets we can characterise them with a mean value and standard deviation. To achieve this, for all the biomarker set we only consider the genes that exist in both the biomarker and also in the breast cancer dataset (note that not every genes in the biomarker set are present in the breast cancer dataset). The four signatures have a mean value and standard deviation of 664.75 and 344.4603 respectively. And for the 44 biomarker genes the mean value and standard deviation is 100.1591 and 120.1567 respectively. There is a major difference between the mean and standard deviation of the two sets.

5.3 Outcome association for the 433 genes

We computed the outcome association of the 433 genes that were reported strictly by the method in the study by Venet et al. 2011. First of all, we extracted the gene expression data of these genes from the breast cancer dataset and we divided the cohorts into two groups and conducted the survival analysis. The p-value we got from the analysis is 0.002546236 which is significant.

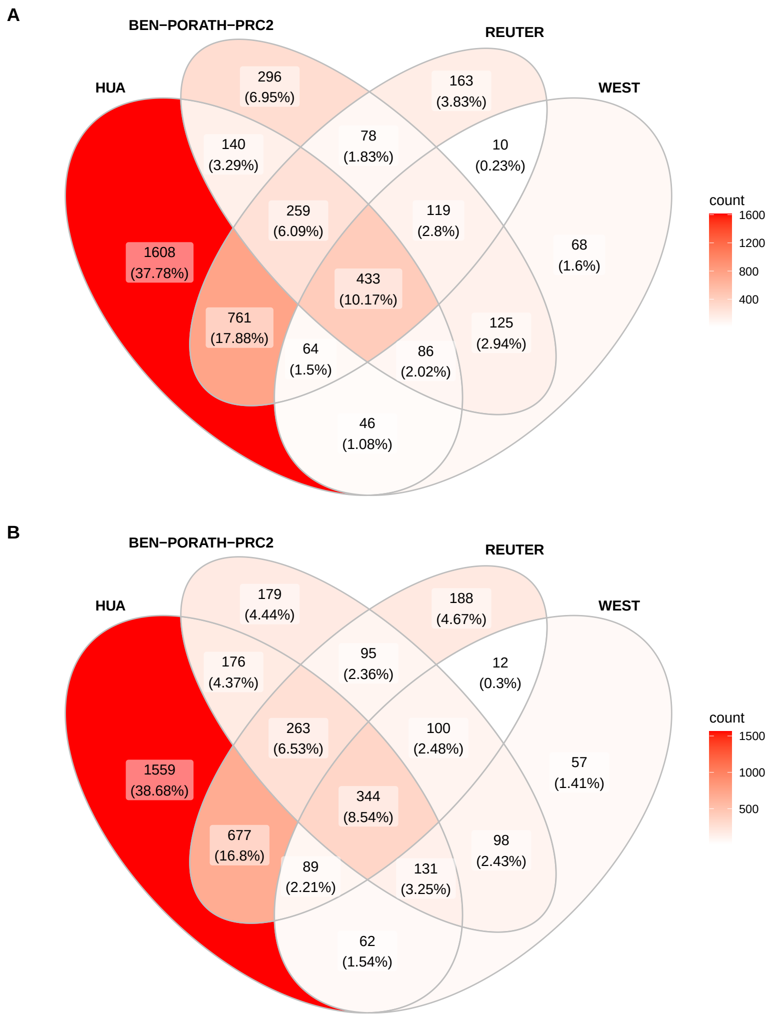

Figure 1: Venn diagrams for the biomarker sets of Hua, Ben-porath-prc2, Reuter and West. A: Overlap for the remaining genes in the signatures. B: Overlap for the remaining GO-terms in the signatures.

	biomarker set	p_{bm}	overlap with 433 genes
1	ABBA	0.85	0
2	ADORNO	1.00	0
3	BEN-PORATH-EXP1	0.85	6
4	BEN-PORATH-PRC2	0.90	0
5	BUESS	0.88	0
6	BUFFA	1.00	0
7	CARTER	0.78	3
8	CHANG	0.81	4
9	CHI	0.76	0
10	CRAWFORD	0.81	7
11	DAI	0.80	0
12	GLINSKY	0.80	0
13	HALLSTROM	0.80	1
14	HE	0.83	0
15	HU	0.75	0
16	HUA	0.89	0
17	IVSHINA	0.64	0
18	KOK	0.81	4
19	KORKOLA	0.95	0
20	LIU	0.81	4
21	MA	0.76	0
22	MILLER	0.71	1
23	MORI	0.69	9
24	PAIK	0.75	0
25	PAWITAN	0.76	1
26	PEI	1.00	0
27	RAMASWAMY	1.00	0
28	REUTER	0.85	0
29	RHODES	0.89	0
30	SAAL	0.87	2
31	SHIPITSIN	0.91	1
32	SORLIE	0.86	1
33	SOTIRIOU-93	0.87	2
34	SOTIRIOU-GGI	0.73	3
35	META-PCNA	0.78	2
36	TAUBE	0.89	2
37	TAVAZOIE	1.00	0
38	VALASTYAN	0.83	0
39	VANTVEER	0.84	0
40	WANG-76	0.85	1
41	WANG-ALK5T204D	0.85	0
42	WELM	1.00	0
43	WEST	0.87	0
44	WHITFIELD	0.83	12
45	WONG-ESC	0.81	6
46	WONG-MITOCHON	0.61	20
47	WONG-PROTEAS	1.00	0
48	YU	0.93	0

Table 145: Shown is how much of the signatures are present in the new gene set G and also the overlap of the signatures with the 433 genes reported earlier.
